# Analysis of Lipophilicity and Pharmacokinetic Parameters of Dipyridothiazine Dimers with Anticancer Potency

**DOI:** 10.3390/pharmaceutics16091235

**Published:** 2024-09-23

**Authors:** Emilia Martula, Beata Morak-Młodawska, Małgorzata Jeleń, Patrick Nwabueze Okechukwu

**Affiliations:** 1 Department of Organic Chemistry, Faculty of Pharmaceutical Sciences in Sosnowiec, The Medical University of Silesia, Jagiellońska 4, 41-200 Sosnowiec, Poland; d201074@365.sum.edu.pl (E.M.); manowak@sum.edu.pl (M.J.); 2 Department of Biotechnology, Faculty of Applied Sciences, UCSI University, Cheras, Kuala Lumpur 56000, Malaysia; patrickn@ucsiuniversity.edu.my

**Keywords:** lipophilicity, isomeric dimers, RP-TLC, ADMET, SAR

## Abstract

Lipophilicity is an essential parameter of a compound that determines the solubility and pharmacokinetic properties that determine the transport of the drug to the molecular target. Dimers of dipyridothiazines are diazaphenothiazine derivatives exhibiting diverse anticancer potential in vitro, which is related to their affinity for histone deacetylase. In this study, the lipophilicity of 16 isomeric dipyridothiazine dimers was investigated theoretically and experimentally by reversed-phase thin-layer chromatography (RP-TLC) in an acetone–TRIS buffer (pH = 7.4). The relative lipophilicity parameter R_M0_ and specific hydrophobic surface area b were significantly intercorrelated, showing congeneric classes of dimers. The parameter R_M0_ was transformed into parameter logP_TLC_ by use of the calibration curve. Molecular descriptors, ADMET parameters and probable molecular targets were determined in silico for analysis of the pharmacokinetic profile of the tested compounds showing anticancer activity. The analyzed compounds were tested in the context of Lipinski’s rule of five, Ghose’s rule and Veber’s rule, confirming their bioavailability.

## 1. Introduction

Lipophilicity is one of the key physicochemical parameters that determines the behavior of a drug in the body [1]. The lipophilicity parameter of drugs has a direct impact on the fate of the drug, i.e., on the ADMET profile, regarding processes such as absorption, distribution, metabolism, excretion and toxicity. When conducting research in the field of drug design, an important issue is the assessment of the lipophilicity of medicinal substances, as it may have a significant impact on their pharmacokinetic properties and toxicity [2]. Numerous publications in the literature indicate that moderate lipophilicity is optimal due to its increased ability to penetrate the cell membrane, which may affect the rate of absorption of the compounds from the gastrointestinal tract or through the skin. Substances with increased lipophilicity can easily penetrate cell membranes and migrate to tissues rich in lipids, which makes it less difficult to reach the molecular target. More lipophilic compounds may be more sensitive to metabolism in the liver via oxidation, reduction and conjugation reactions. Therefore, lipophilicity also influences pharmacological activity and toxicity [3,4,5,6,7,8]. Lipinski’s rule of five or Ghose’s rule emphasizes the importance of lipophilicity in the drug design process [9,10]. Currently, theoretical and experimental methods are commonly used to determine lipophilicity parameters. In silico methods are used to estimate the lipophilicity parameter expressed as the decimal logarithm of the partition coefficient P (logP). The broad development of cheminformatics has influenced, over the last 20 years, the number of programs available for in silico prediction of this important parameter and other ADMET properties that can be estimated on ADMETlab, SwissADME, PreADMET or MetaTox servers [11,12]. Computational approaches are useful for the rapid prediction of logP values, especially in the early stages; however, the obtained results should always be verified experimentally. Among the experimental methods for determining lipophilicity, the most prominent are chromatographic methods, i.e., reversed-phase thin-layer chromatography (RP-TLC) and high-performance liquid chromatography (HPLC). These methods have replaced the expensive and environmentally unfriendly shake-flask method, which allowed for the determination of lipophilicity only in the range of −2 to 4 [13,14,15].

Phenothiazines are active heterocycles that are widely used in psychiatry as neuroleptic drugs. Their lipophilicity being within the limits logP = 5–6 allows them to penetrate the blood–brain barrier and interact with D2 receptors, which determine antipsychotic activity [16,17]. Dipyridothiazines belong to a group of modified phenothiazines that have pyridine rings in their structure instead of benzene rings. These compounds show promising anticancer activities, as well as immunomodulatory activities. Investigation of the mechanism of anticancer activity of these compounds showed the induction of cell death through oxidative damage to cell components. PCR Array studies of the apoptosis pathway showed the internal (mitochondrial) and external (receptor) apoptosis pathway, while the cell cycle analysis showed the cytostatic effect of the tested compounds and significant inhibition of the cell cycle in the G2/M phase [18]. Previous studies showed that their dipyridothiazine derivatives have moderate lipophilicity in the range of 1–4, which to some extent correlates with their activity [18,19,20]. Recently, we described new dipyridothiazine dimers in which two dipyridothiazine units (1,6-, 1,8-, 2,7- and 3,6-diazaphenothiazine) were linked with selected linkers *o*-, *m*-, *p*-xylene and 2,6-dimethylpyridine (lutidine) (**1a,b,c,d–4a,b,c,d**) [21,22]. These compounds were tested for cytotoxicity towards breast cancers (MCF7, MDA-MB-231); colon cancers (SW480, SW620); lung cancer (A-549); glioblastoma (LN-229); as well as normal muscle cells (L6) and keratinocytes (HaCaT). Reference compounds such as doxorubicin and cisplatin were used in the studies. The tested compounds were non-toxic at the tested concentrations in relation to normal cells. Various results of anticancer activity were obtained IC_50_ in the range of 0.1–100 μM. Most of the dimers tested were characterized by significant anticancer activity against breast cancer line MCF7 (IC_50_ = 1.14 µM) and colon cancer SW480 (IC_50_ = 3.11 µM), giving highly promising results. Dimer derivatives with the *m*-xylene linker (**1b–4b**) showed the lowest anticancer activity in this group of compounds, which was explained using molecular docking in relation to histone deacetylase (HDAC4). These compounds, unlike other derivatives, bound in a different place of the receptor, which resulted from their conformation [22].

**Figure 1:**
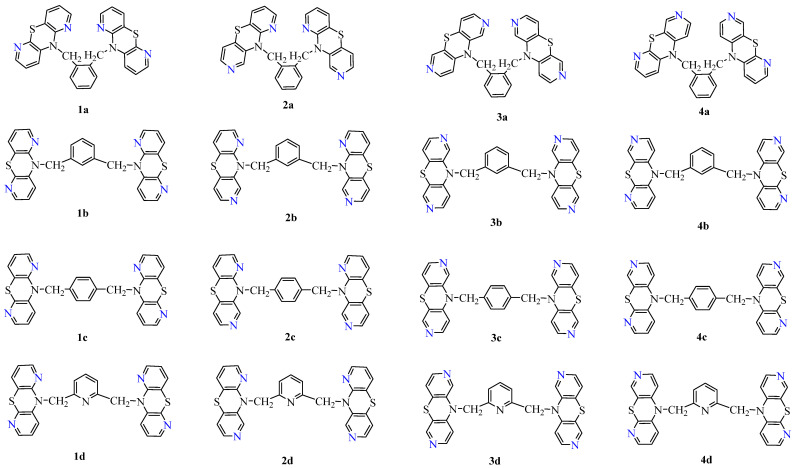
The structure of the sixteen tested compounds (**1a,b,c,d**–**4a,b,c,d**).

The aim of this work was to investigate the lipophilicity of new dipyridithiazine dimers (**1a,b,c,d–4a,b,c,d**,) determined experimentally by the RP-TLC method and using calculated computer programs to determine the ADMET parameters and to search for the relationship between their lipophilicity, structure and biological activity. Lipophilicity studies were conducted with the hope of gaining deeper insight into differences in biological activity. The structure of the sixteen tested compounds (**1a,b,c,d–4a,b,c,d**) is shown in Figure 1.

## 2. Materials and Methods

### 2.1. Reagents

Synthesis, purification and full structural analysis of new compounds **1a,b,c,d–4a,b,c,d** have been described [21,22].

The following was used to prepare the mobile phase: acetone (POCh, Gliwice, Poland) and buffer TRIS (tris(hydroxymethyl)aminomethane, Fluka). The calibration curve was created using the following five different chemical compounds (standards) for which lipophilicity parameters were described (logP_lit._): benzamide **I** (Fluka, Buchs, Switzerland), acetanilide **II** (POCh, Gliwice, Poland), acetophenone **III** (POCh, Gliwice, Poland), 4-bromoacetophenone **IV** (Fluka, Buchs, Switzerland) and benzophenone **V** (Fluka, Buchs, Switzerland).

### 2.2. Chromatographic Procedure

The RP-TLC method was used to determine the experimental lipophilicity in accordance with information from the literature [20]. The stationary phase used was the modified silica gel RP 18F254S (Merck, Darmstad, Gerrmany). RP-TLC plates with dimensions were prepared 5 cm × 10 cm. A starting line was marked 1 cm from the edge of the plate, to which solutions of the tested compounds (**1a,b,c,d–4a,b,c,d**) and standards **I–V** at a concentration of 2 μM/mL were applied. So, the prepared plates were transferred to a chromatographic chamber saturated with vapors of acetone solution and aqueous buffer TRIS (0.2 M, buffer pH = 7.4). Solutions with a volume of 25 mL were used in the ratios acetone–Tris buffer in the following proportions: 50:50, 55:45, 60:40, 65:35 and 70:30. The measurements were repeated three times. The spots were observed under UV light at a wavelength of 254 nm. The R_F_ values were calculated for each spot. Based on the R_F_ value, the R_M_ value was determined according to the following formula:

R_M_ = log(1/R_F_ − 1) (1)

Next, a graph of the relationship between the acetone concentration and the R_M_ value was plotted for each compound. Extrapolation to zero acetone concentration allowed us to obtain the values of the relative lipophilicity parameter R_M0_, which is an indicator of the partition between the non-polar stationary phase and the polar mobile phase. This was achieved according to the following mathematical equation:

R_M_ = R_M0_ + bC (2)

where C is the volume fraction of the organic modifier, i.e., acetone in the mobile phase, and b is the change in the R_M_ value as a result of a 1% increase in the organic modifier in the mobile phase (related to the hydrophobic specific surface).

### 2.3. Theoretical Lipophilicity, ADMET Parameters and Target Prediction

The calculated lipophilicity was determined using various web servers, including VCCLAB [23], ChemDraw [24], SwissADME [25], and using iLOGP, XLOGP3, WLOGP, MLOGP, SILICOS-IT, LogP ChemDraw, Mol inspiration and Alogps calculated models. The molecular descriptor and ADME parameters were calculated using the SwissADME and PreADME servers [25,26]. Target prediction was determined by the SwissTargetPrediction server [27].

## 3. Results

The study of lipophilicity parameters began with theoretical calculations of lipophilicity parameters (logP_calcd._). Lipophilicity calculations were performed using eight different mathematical modules that are available on the popular and widely used internet servers VCCLAB [23], SwissADME [25] and ChemDraw [24]. The obtained results are included in Table 1. It can be assumed that these results are only estimated results, because, depending on the calculation module used, the obtained analyses were with a large variability, which reached even more than two units.

Then, experimental tests of the lipophilicity coefficient were carried out using reversed-phase RP-TLC chromatography. At the beginning, the R_F_ parameter of the tested dimers (**1a,b,c,d–4a,b,c,d**) was measured, which was converted into the R_M_ parameter according to Equation (1).

R_M_ values decreased linearly with increasing acetone concentration in the mobile phase (r = 0.9562–0.9973). Extrapolation to zero acetone concentration allowed us to obtain the values of the relative lipophilicity parameter R_M0_ (Equation (2)). The R_M0_ values of dipirydothiazine dimers are in the range of 0.8993–3.5760 (Table 2).

Then, a calibration curve was created under the same measurement conditions, allowing the relative lipophilicity parameter R_M0_ to be converted to logP_TLC_. Five reference substances (**I–V**) with the literature logP_lit_ were used in a wide range of the logP parameter 0.62–4.45 (Table 3).

The standard curve equation prepared with standard substances **I–V** is presented below as follows:

logP_TLC_ = 0.839R_M0_ + 0.0514   (r = 0.9911) (3)

The calibration curve made it possible to convert the value of the relative lipophilicity parameter R_M0_ of the tested dimers into the value of the absolute lipophilicity parameter logP_TLC_. The logP_TLC_ values for all dimers (**1a,b,c,d–4a,b,c,d**) are presented in Table 4.

In parallel with the experimental research, analyses of molecular descriptors and Lipinski’s, Ghose’s and Veber’s parameters were carried out using the SwissADME server (Table 5) [25]. The full set of data is included in the Supplementary Materials.

In computational studies, these compounds do not show significant differences in molecular descriptors due to the fact that they are isomeric molecules (Table 5), but they do show differences in ADMET parameters (Table 6) [26].

Among in silico ADMET parameters, the Caco-2 [30] and MDCK [31] cell models were indicated, allowing for the prediction of the absorption of oral drugs. The human intestinal absorption (HIA) model allows for the prediction and identification of drug candidates; also for oral use, the skin permeability (SP) model can predict and identify potential drugs for oral delivery and transdermal delivery. Penetration of the blood–brain barrier (BBB) can provide information about a drug with the ability to cross the blood–brain barrier and act in the central nervous system (CNS), while the plasma protein binding (PPB) model provides information on its distribution [26,32]. The experimental R_M0_ values were correlated with ADMET activities (Table 7).

Using the SwissTargetPrediction server [27], probable molecular targets for the described compounds were identified. The results of molecular targets with the highest probability index are summarized in Table 8. The full results of this part are included in the Supplementary Materials.

## 4. Discussion

This study focuses on the analysis of the physicochemical properties of new diazaphenothiazine dimers exhibiting diverse anticancer activity. These compounds were created by combining two units of selected dipyridothiazines (1,6-, 1,8-, 2,7- and 3,6-diazaphenothiazines) with selected *o*-, *m*-, *p*-xylene and 2,6-dimethylpyridine (lutidine) linkers (Figure 1). In in vitro studies, these compounds showed significant anticancer activity against breast and colon cancer lines, and, more importantly, low cytotoxicity against normal skin and muscle cell lines [21,22].

**Figure 2:**
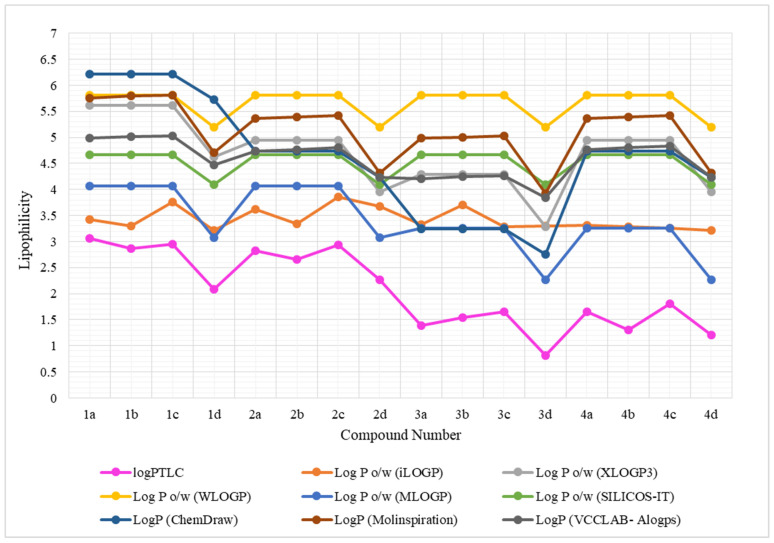
Graphical visualization of calculated logP values of the tested compounds with comparison of logP_TLC_ (indicated in pink).

The use of computational programs resulted in different logP_calcd_ values depending on the computational module used and the dimer structure. Among the eight computational programs, five programs (XLOGP3, WLOGP, MLOGP, SILCOS-IT and logP (ChemDraw, version 16.0) did not distinguish between isomeric dimers containing *o*-, *m*- and *p*-xylene fragments in different combinations of dipyridothiazines, giving the same numerical values of the logP_calcd_ parameter. The remaining computational models, i.e., iLOGP LogP(Molinspiration) and LogP(VCCLAB), provided different values of the lipophilicity parameter for the tested dimers. The iLOGP program indicated that the lowest lipophilicity (logP_calcd_ = 3.22) is characterized by dimers **1d** and **4d**, composed of two units of 1,6-diazaphenothiazine and 3,6-diazaphenothiazine, respectively, connected with a 2,6-dimethylpyridine linker, while the highest lipophilicity (logP_calcd_ = 3.76) was indicated for dimer **1c** containing 1,6-diazaphenothiazine in its structure and *p*-xylene linker. The calculation modules LogP (Molinspiration) and LogP (VCCLAB) indicated the highest lipophilicity parameter for the **1c** dimer (logP_calcd._ = 5.81 and logP_calcd_ = 5.03, respectively), too, while the lowest parameters were determined by these programs for the compound **3d** (logP_calcd_ = 3.93 and logP_calcd_ = 3.84, respectively). Analyzing the calculation data included in Table 1, it can be seen that the logP_calcd_ values for the same compound differed significantly, reaching the largest differences of up to two units on a logarithmic scale. The results of these computational analyses are graphically presented for individual compounds in Figure 2. The obtained results again indicated the need to perform experimental measurements in order to correctly and accurately determine the lipophilicity parameter.

The experimental values of relative lipophilicity R_M0_ showed that dimer **1a**, composed of two 1,6-diazaphenothiazine units connected with an *o*-xylene fragment, is the most lipophilic, while dimer **3d**, composed of two 2,7-diazaphenothiazine units connected with a 2,6-dimethylpyridine fragment, is the least lipophilic.

It is known from the literature [33,34,35] that the R_M0_ parameter and the specific surface area b, being significantly correlated, allow us to show chromatographic similarity in the groups of tested compounds. This allows us to determine the class of congenericity, i.e., the chromatographic similarity of the tested compounds. The mutual correlation between the two parameters R_M0_ and b for all tested dimers gave an equation with a moderate correlation coefficient, which depends on the type of linker connecting the dipyridothiazine units, as follows:

R_M0_ = −93.328b − 0.5209   (r = 0.9886)

and showing the presence of the expected congeneric subclasses, as follows:

- Dimers **1a–4a** *R*_M0_ = −89.395*b* − 0.4208 (r = 0.9952);
- Dimers **1b–4b** *R*_M0_ = −106.55*b* − 1.1075 (r = 0.9957);
- Dimers **1c–4c** *R*_M0_ = −93.8444*b* − 0.5607 (r = 0.9912);
- Dimers **1d–4d** *R*_M0_ = −96.341*b* − 0.5183607 (r = 0.9933).

In the next stage of the research, the absolute lipophilicity parameter of logP_TLC_ was determined. For this purpose, a calibration curve was created, allowing the relative lipophilicity parameter of R_M0_ to be converted to logP_TLC_. In this process, the following reference substances with a known lipophilicity parameter logP were used: benzamide, acetanilide, acetophenone, 4-bromoacetophenone and benzophenone (Table 3).

**Figure 3:**
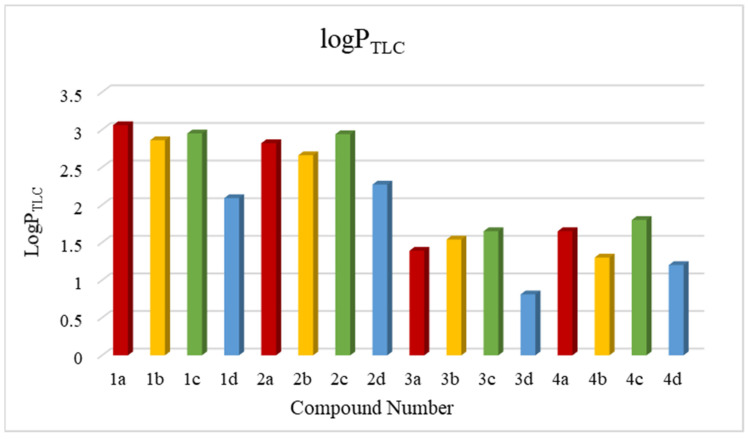
Graphical visualization of the logP_TLC_ values of the tested dipyridothiazine dimers.

Using the calibration curve equation, the relative lipophilicity parameter R_M0_ was converted into absolute logP values, which are listed in Table 4. They are in the range of 0.81–3.06 (Table 4). Dimer **3d** has the lowest parameter and the highest parameter has the derivative **1a**. These values differ significantly from the computer-calculated parameters, which are also shown in Figure 3.

The obtained results depend on the type of linker connecting the dipyridothiazine units and the structure of the dipyridothiazine unit, in particular, the position of the nitrogen atoms. Among the tested dimers, significantly higher lipophilicity parameters were achieved by those containing 1,6-diaza- and 1,8-diazaphenothiazines in their structure **1a–2d**. Both groups of derivatives have a nitrogen atom in position 1, in the immediate vicinity of the thiazine nitrogen atom. However, lower lipophilicity parameters are demonstrated by dimers with 2,7-diaza- and 3,6-diazphenothiazine units **3a–4d**, in which the nitrogen atoms are essentially close to the sulfur atom.

Looking at lipophilicity in the light of the anticancer activity, it should be noted that the most anticancer active **4d** dimer (IC_50_ < 0.1 μM in relation to MCF-7 breast cancer cells) has the low lipophilicity logP = 1.21 in the tested group of compounds. Dimer **1d** (IC_50_ = 1.14 μM in relation to MCF7 cancer cells) showed equally high anticancer activity, with higher activity with logP = 2.09. In the group of dimers subjects, there were also dimers with an *m*-xylene moiety, which showed very weak cytotoxic effects with an IC_50_ > 60 μM and had various lipophilicity in the range of logP = 1.30–2.86. Previous studies [22] on quantum mechanical calculations and molecular docking explained the differences in the anticancer activity and the inactivity of isomers with the *m*-xylene system **1b–4b** and the 2,6-lutidine system **1d–4d**, indicating different binding sites in relation to histone deacetylase. Additionally, these lipophilicity studies show that this parameter is not a factor influencing the anticancer effects. Nevertheless, it is one of the foundational factors determining the transport and fate of a chemical substance in the body. The tested dimers were analyzed for molecular descriptors, which include molecular mass, number of sites that are hydrogen acceptors and donors, number of rotating bonds, molar refraction and TPSA surface, which allowed checking the requirements for meeting Lipinski’s rule of five, Ghose’s rule and Veber’s rule (Table 5). All tested derivatives meet the requirements of Lipinski’s rule of five and Veber’s rule. These results indicate that the tested derivatives may become drugs with the properties of an orally active drug. In experimental studies, dimers showed lipophilicity lower than five, which indicates that they are better able to migrate through protein–lipid membranes, and thus there is a small chance of these derivatives becoming stuck in lipid zones.

However, it can be noted that the tested dimers do not meet the requirements of Ghose’s rules, which restrictively indicate that the molecular weight must be within the range of 160 to 480 g/mol [25], which, however, does not disqualify the tested compounds, as they are known medicinal substances used in anticancer therapies, the molecular weights of which are substantially higher.

In the presented project, analyses of ADMET parameters (Table 6) were performed for the tested isomeric dimers using the popular PreADMET internet server [26,30,31,32]. The tested dimers show a moderate ability to penetrate the blood–brain barrier, which can be considered an advantage, as there is a low probability of side effects related to the central nervous system. The BBB penetration index is in the range of 0.2 to 1.7 and depends on the type of dipyridothiazine building a specific dimer. Caco-2 cell permeability was diverse and dependent on both the dipyridothiazine fragment and the linker building the dimer. All tested compounds showed a high HIA index, which ranged from 97 to 98. MDCK cell permeability was low and ranged from 0.05 to 4. These values differ significantly in the study group compounds and depend on the structure of the dimer. Similar differences were observed for the PPB parameter. Table 6 also includes the calculated parameters of the reference drug doxocubicin, for comparison. When comparing the ADMET properties of the tested dimers with the reference drug, large differences can be noticed, which can be explained by structural differences in the compounds. ADMET properties such as Caco-2 permeability, SP, BBB, HIA, MDCK and PPB were correlated with the lipophilicity parameter R_M0_ of the tested dimers. The correlation results obtained were very diverse, and are presented in Table 7. Good correlations were found for Caco-2, PPB, HIA and BBB with r = 0.7288, 0.6918 and 0.6705, and 0.6158. A weak correlation was obtained for the MDCK and SP with r = 0.3103, 0.3022. It is worth noting that all correlations are described by third-degree equations, which may be an indication that lipophilicity is one of many factors affecting ADMET parameters.

Finally, molecular targets were determined for the tested dimers using the SwissTargetPredicion server. Table 8 contains the results obtained for molecular cells with the highest probability indexes, and all the results are collected in the Supplementary Materials. These studies both confirmed the anticancer potential and allowed for the identification of other molecular targets for the tested dimers. The tested derivatives may affect the activity of kinases, CG and AG family proteins, proteases, cytochrome 450 and phosphodiesterase. The obtained results are an indication for further research at the in vitro level.

## 5. Conclusions

To sum up, the lipophilicity of sixteen new antiproliferative dipyridothiazine dimers with *o*-, *m*-, *p*-xylene and 2,6-lutidine linkers was determined both by computational models and experimentally using RP-TLC. The experimental RP-TLC showed that these compounds exhibit moderate lipophilicity. None of the calculation programs reported logP_calcd_ values similar to logP_TLC_ values, which can be attributed to their specific, non-flat structure.

The tested derivatives meet the Lipinski rule, which means they can become drugs when administered orally. Structural analysis in terms of lipophilicity showed that this parameter is influenced by both the fragment connecting two dipyridothiazine units and the location of nitrogen atoms. Analyses of the relationship between ADMET properties and lipophilicity were performed, presenting preliminary SAR results. The obtained results are valuable and useful for further pharmacological studies of this group of dipyridothiazine derivatives.

## Figures and Tables

**Table 1:** The calculated lipophilic parameters logP_calcd._ for dimers **1a,b,c,d–4a,b,c,d** using the internet databases SwissADME, ChemDraw and VCCLAB.

No.	LogP_calcd._							
	iLOGP	XLOGP3	WLOGP	MLOGP	SILICOS-IT	LogP (ChemDraw)	LogP (Mol Inspiration)	LogP (VCCLAB Alogps)
**1a**	3.42	5.62	5.81	4.07	4.67	6.22	5.76	4.98
**1b**	3.30	5.62	5.81	4.07	4.67	6.22	5.79	5.01
**1c**	3.76	5.62	5.81	4.07	4.67	6.22	5.81	5.03
**1d**	3.22	4.62	5.20	3.07	4.10	5.73	4.71	4.47
**2a**	3.62	4.95	5.81	4.07	4.67	4.73	5.37	4.74
**2b**	3.34	4.95	5.81	4.07	4.67	4.73	5.39	4.76
**2c**	3.85	4.95	5.81	4.07	4.67	4.73	5.42	4.80
**2d**	3.67	3.95	5.20	3.07	4.10	4.24	4.32	4.24
**3a**	3.33	4.29	5.81	3.26	4.67	3.24	4.98	4.20
**3b**	3.70	4.29	5.81	3.26	4.67	3.24	5.00	4.25
**3c**	3.29	4.29	5.81	3.26	4.67	3.24	5.03	4.26
**3d**	3.30	3.29	5.20	2.26	4.10	2.75	3.93	3.84
**4a**	3.31	4.95	5.81	3.26	4.67	4.73	5.37	4.77
**4b**	3.28	4.95	5.81	3.26	4.67	4.73	5.39	4.81
**4c**	3.26	4.95	5.81	3.26	4.67	4.73	5.42	4.84
**4d**	3.22	3.95	5.20	2.26	4.10	4.24	4.32	4.24

**Table 2:** The R_M0_ values and b (slope) and r (correlation coefficient) of equation R_M_ = R_M0_ + bC for compounds **1a,b,c,d–4a,b,c,d**.

No.	−b	R_M0_	r
**1a**	0.045	3.5760	0.9973
**1b**	0.0426	3.3468	0.9806
**1c**	0.0432	3.4546	0.9979
**1d**	0.0300	2.4253	0.9978
**2a**	0.0409	3.3041	0.9983
**2b**	0.0385	3.1117	0.9850
**2c**	0.0418	3.4411	0.9972
**2d**	0.0327	2.6375	0.9941
**3a**	0.0217	1.6016	0.9856
**3b**	0.0274	1.7742	0.9674
**3c**	0.0252	1.9040	0.9850
**3d**	0.0139	0.8993	0.9562
**4a**	0.0274	1.9033	0.9933
**4b**	0.0243	1.4866	0.9621
**4c**	0.0296	2.0767	0.9983
**4d**	0.0209	1.3580	0.9765

**Table 3:** R_M0_ and logP_lit_ values and b (slope) and r (correlation coefficient) of equation R_M_ = R_M0_ + bC for standards **I–V.**

Parameters	I	II	III	IV	V
**LogP_lit._**	0.64 [28]	1.21 [29]	1.58 [29]	2.43 [29]	4.45 [28]
**R_M0_**	0.5858	0.9275	1.5099	2.1803	2.6378
**−b**	0.0168	0.0181	0.0225	0.0288	0.0346
**r**	0.9954	0.9936	0.9920	0.9960	0.9930

**Table 4:** The logP_TLC_ values of investigated dimers **1a,b,c,d–4a,b,c,d.**

No.	1a	1b	1c	1d	2a	2b	2c	2d	3a	3b	3c	3d	4a	4b	4c	4d
logP_TLC_	3.06	2.86	2.95	2.09	2.82	2.66	2.94	2.27	1.39	1.54	1.68	0.81	1.65	1.30	1.80	1.21

**Table 5:** The molecular descriptor and parameters of Lipinski’s, Ghose’s and Veber’s rules for investigated compounds calculated using SwisADME server [25].

No.	Molecular Mass (g/mol)	H-Bond Acceptors	H-Bond Donors	Rotatable Bonds	Molar Refractivity	TPSA [Å^2^]	P-gp Substrate	Lipinski’s Rules	Ghose’s Rules	Veber’s Rules	Muegge’s Rules
**1a**	504.63	4	0	4	149.60	108.64	+	+	−	+	−
**1b**	504.63	4	0	4	149.60	108.64	+	+	−	+	−
**1c**	504.63	4	0	4	149.60	108.64	+	+	−	+	−
**1d**	505.62	5	0	4	147.40	121.53	+	+	−	+	+
**2a**	504.63	4	0	4	149.60	108.64	+	+	−	+	+
**2b**	504.63	4	0	4	149.60	108.64	+	+	−	+	+
**2c**	504.63	4	0	4	149.60	108.64	+	+	−	+	+
**2d**	505.62	5	0	4	147.40	121.53	+	+	−	+	+
**3a**	504.63	4	0	4	149.60	108.64	+	+	−	+	+
**3b**	504.63	4	0	4	149.60	108.64	+	+	−	+	+
**3c**	504.63	4	0	4	149.60	108.64	+	+	−	+	+
**3d**	505.62	5	0	4	147.40	121.53	+	+	−	+	+
**4a**	504.63	4	0	4	149.60	108.64	+	+	−	+	+
**4b**	504.63	4	0	4	149.60	108.64	+	+	−	+	+
**4c**	504.63	4	0	4	149.60	108.64	+	+	−	+	+
**4d**	505.62	5	0	4	147.40	121.53	+	+	−	+	+

+ = meeting the rules, − = not meeting the rules.

**Table 6:** The predicted ADME activities using PreADMET server [26].

No.	Caco-2 Permeability (nm/s)	Skin Permeability (SP, log Kp)	BBB Permeability (C.brain/C.blood)	HIA (%)	MDCK (nm/s)	Plasma Protein Binding (PPB,%)
**1a**	35.7262	−2.60631	1.783	97.816	0.275	100
**1b**	30.5877	−2.62873	0.208	97.816	0.073	100
**1c**	32.9405	−2.63079	0.202	97.816	3.944	100
**1d**	28.8091	−2.91305	0.415	98.019	0.205	98
**2a**	29.7800	−2.91532	0.905	97.816	0.1395	96
**2b**	26.4756	−2.94119	0.224	97.816	0.0607	97
**2c**	27.9283	−2.94361	0.311	97.816	1.399	95
**2d**	25.5742	−3.25587	0.205	98.019	0.114	91
**3a**	29.3153	−3.48028	0.391	97.816	0.151	91
**3b**	25.9114	−3.50753	0.400	97.816	0.064	90
**3c**	27.4437	−3.5101	0.201	97.816	1.636	90
**3d**	25.1325	−3.81394	0.183	98.019	0.121	87
**4a**	32.0035	−3.15846	0.258	97.816	4.062	97
**4b**	29.4980	−3.1559	0.938	97.816	0.072	99
**4c**	32.0035	−3.15846	0.258	97.816	4.062	97
**4d**	28.0612	−3.47613	0.403	98.019	0.203	91
**Doxorubicin**	17.7263	−4.73786	0.036	56.841	1.204	31

**Table 7:** The correlation of the R_M0_ values with predicted ADMET activities for compounds **1a,b,c,d–4a,b,c,d**.

No. of Compounds	ADMET Activities	Equation	r
**1a,b,c,d–** **4a,b,c,d**	**Caco-2**	Caco-2 = 4.0763 R_M0_^3^ − 26.748 R_M0_^2^ + 54.697 R_M0_ − 6.1633	0.7288
**1a,b,c,d–** **4a,b,c,d**	**SP**	SP = 0.5685 R_M0_^3^ − 3.7459 R_M0_^2^ + 7.621 R_M0_ − 7.7475	0.3022
**1a,b,c,d–** **4a,b,c,d**	**BBB**	BBB = 0.4934 R_M0_^3^ − 3.0831 R_M0_^2^ + 5.8357 R_M0_ − 2.9445	0.6158
**1a,b,c,d–** **4a,b,c,d**	**HIA**	HIA = −0.118 R_M0_^3^ + 0.7997 R_M0_^2^ − 1.7015 R_M0_ + 99.01	0.6705
**1a,b,c,d–** **4a,b,c,d**	**MDCK**	MDCK = 0.7955 R_M0_^3^ − 5.8698 R_M0_^2^ + 13.621 R_M0_ − 8.7393	0.3143
**1a,b,c,d–** **4a,b,c,d**	**PPB**	PPB = −2.0899 R_M0_^3^ − 14.54 R_M0_^2^ + 34.436 R_M0_ + 66.883	0.6918

**Table 8:** Molecular targets indicated using SwissTargetPrediction server [27].

No. of Compound	Target Prediction		
**1a**	Kinase	Family C G protein-coupled receptor	Phosphodiesterase
**1b**	Ligand-gated ion channel	Cytochrome P450	Phosphodiesterase
**1c**	Kinase	Protease	Enzyme
**1d**	Kinase	Family C G protein-coupled receptor	Phosphodiesterase
**2a**	Kinase	Enzyme	Phosphodiesterase
**2b**	Kinase	Histone deacetylase 1	Phosphodiesterase
**2c**	Kinase	Family C G protein-coupled receptor	Protease
**2d**	Kinase	Family C G protein-coupled receptor	Phosphodiesterase
**3a**	Kinase	Cytochrome P450	Protease
**3b**	Cytochrome P450	Protease	Phosphodiesterase
**3c**	Kinase	Enzyme	Protease
**3d**	Reader	Family C G protein-coupled receptor	Protease
**4a**	Phosphodiesterase	Family C G protein-coupled receptor	Protease
**4b**	Kinase	Family A G protein-coupled receptor	Cytochrome P450
**4c**	Kinase	Family C G protein-coupled receptor	Voltage-gated ion channel
**4d**	Phosphodiesterase	Bromodomain-containing protein 4,3,2	Cytochrome P450

## Data Availability

Data are available from the authors.

## Supplementary Materials

The following supporting information can be downloaded at: https://www.mdpi.com/article/10.3390/pharmaceutics16091235/s1.
