# Supplementary material for: Analysis of Lipophilicity and Pharmacokinetic Parameters of Dipyridothiazine Dimers with Anticancer Potency
Source: Pharmaceutics. 2024 Sep 23;16(9):1235. doi: 10.3390/pharmaceutics16091235 (PMC11435374; doi:10.3390/pharmaceutics16091235)

Supplementary Material

Analysis of Lipophilicity and Pharmacokinetic Parameters of Dipyridothiazine Dimers with Anticancer Potency

Emilia Martula [1], Beata Morak-Młodawska [1,*], Małgorzata Jeleń [1] and Patrick Nwabueze Okechukwu [2]

[1] Department of Organic Chemistry, Faculty of Pharmaceutical Sciences in Sosnowiec, The Medical University of Silesia, Jagiellońska 4, 41-200 Sosnowiec, Poland; d201074@365.sum.edu.pl (E.M.); manowak@sum.edu.pl (M.J.)
[2] Department of Biotechnology, Faculty of Applied Sciences, UCSI University, Cheras, Kuala Lumpur 56000, Malaysia; patrickn@ucsiuniversity.edu.my
* Correspondence: bmlodawska@sum.edu.pl

Content

1. ADME analysis results and probability of molecular targets of compound **1a** **2**
2. ADME analysis results and probability of molecular targets of compound **1b** **3**
3. ADME analysis results and probability of molecular targets of compound **1c** **4**
4. ADME analysis results and probability of molecular targets of compound **1d** **5**
5. ADME analysis results and probability of molecular targets of compound **2a** **6**
6. ADME analysis results and probability of molecular targets of compound **2b** **7**
7. ADME analysis results and probability of molecular targets of compound **2c** **8**
8. ADME analysis results and probability of molecular targets of compound **2d** **9**
9. ADME analysis results and probability of molecular targets of compound **3a** **10**
10. ADME analysis results and probability of molecular targets of compound **3b** **11**
11. ADME analysis results and probability of molecular targets of compound **3c** **12**
12. ADME analysis results and probability of molecular targets of compound **3d** **13**
13. ADME analysis results and probability of molecular targets of compound **4a** **14**
14. ADME analysis results and probability of molecular targets of compound **4b** **15**
15. ADME analysis results and probability of molecular targets of compound **4c** **16**
16. ADME analysis results and probability of molecular targets of compound **4d** **17**

ADME analysis results and probability of molecular targets of compound **1a**

Molecule 1

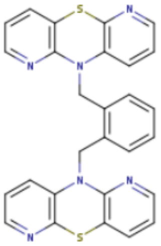

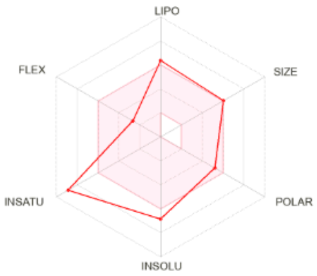

SMILES c1ccc(c(c1)CN1c2ncccc2Sc2c1cccn2)CN1c2ncccc2Sc2c1cccn2

Physicochemical Properties	
Formula	C28H20N6S2
Molecular weight	504.63 g/mol
Num. heavy atoms	36
Num. arom. heavy atoms	30
Fraction Csp3	0.07
Num. rotatable bonds	4
Num. H-bond acceptors	4
Num. H-bond donors	0
Molar Refractivity	149.60
TPSA	108.64 Å²

Lipophilicity	
Log $P_{o/w}$ (iLOGP)	3.42
Log $P_{o/w}$ (XLOGP3)	5.62
Log $P_{o/w}$ (WLOGP)	5.81
Log $P_{o/w}$ (MLOGP)	4.07
Log $P_{o/w}$ (SILICOS-IT)	4.67
Consensus Log $P_{o/w}$	4.72

Water Solubility	
Log *S* (ESOL)	-6.86
Solubility	6.93e-05 mg/ml ; 1.37e-07 mol/l
Class	Poorly soluble
Log *S* (Ali)	-7.66
Solubility	1.09e-05 mg/ml ; 2.17e-08 mol/l
Class	Poorly soluble
Log *S* (SILICOS-IT)	-10.48
Solubility	1.67e-08 mg/ml ; 3.30e-11 mol/l
Class	Insoluble

Pharmacokinetics	
GI absorption	Low
BBB permeant	No
P-gp substrate	Yes
CYP1A2 inhibitor	No
CYP2C19 inhibitor	Yes
CYP2C9 inhibitor	Yes
CYP2D6 inhibitor	No
CYP3A4 inhibitor	Yes
Log K_p (skin permeation)	-5.39 cm/s

Druglikeness	
Lipinski	Yes; 1 violation: MW>500
Ghose	No; 3 violations: MW>480, WLOGP>5.6, MR>130
Veber	Yes
Egan	Yes
Muegge	No; 1 violation: XLOGP3>5
Bioavailability Score	0.55

Medicinal Chemistry	
PAINS	0 alert
Brenk	0 alert
Leadlikeness	No; 2 violations: MW>350, XLOGP3>3.5
Synthetic accessibility	3.98

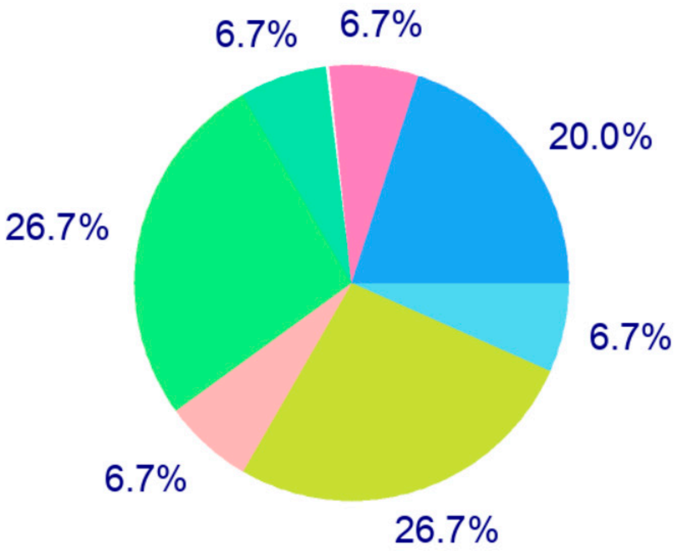

ADME analysis results and probability of molecular targets of compound **1b**

Molecule 1

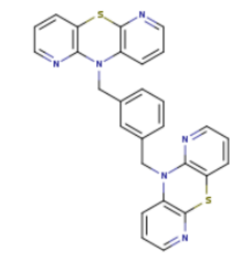

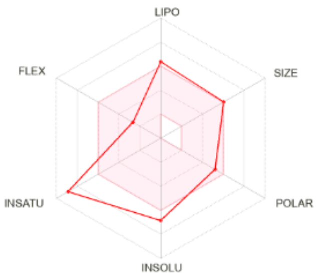

SMILES c1cc(cc(c1)CN1c2ncccc2Sc2c1cccn2)CN1c2ncccc2Sc2c1cccn2

Physicochemical Properties	
Formula	C28H20N6S2
Molecular weight	504.63 g/mol
Num. heavy atoms	36
Num. arom. heavy atoms	30
Fraction Csp3	0.07
Num. rotatable bonds	4
Num. H-bond acceptors	4
Num. H-bond donors	0
Molar Refractivity	149.60
TPSA	108.64 Å²
**Lipophilicity**	
Log $P_{o/w}$ (iLOGP)	3.30
Log $P_{o/w}$ (XLOGP3)	5.62
Log $P_{o/w}$ (WLOGP)	5.81
Log $P_{o/w}$ (MLOGP)	4.07
Log $P_{o/w}$ (SILICOS-IT)	4.67
Consensus Log $P_{o/w}$	4.69

Water Solubility	
Log *S* (ESOL)	-6.86
Solubility	6.93e-05 mg/ml ; 1.37e-07 mol/l
Class	Poorly soluble
Log *S* (Ali)	-7.66
Solubility	1.09e-05 mg/ml ; 2.17e-08 mol/l
Class	Poorly soluble
Log *S* (SILICOS-IT)	-10.48
Solubility	1.67e-08 mg/ml ; 3.30e-11 mol/l
Class	Insoluble
**Pharmacokinetics**	
GI absorption	Low
BBB permeant	No
P-gp substrate	Yes
CYP1A2 inhibitor	No
CYP2C19 inhibitor	Yes
CYP2C9 inhibitor	Yes
CYP2D6 inhibitor	No
CYP3A4 inhibitor	Yes
Log K_p (skin permeation)	-5.39 cm/s
**Druglikeness**	
Lipinski	Yes; 1 violation: MW>500
Ghose	No; 3 violations: MW>480, WLOGP>5.6, MR>130
Veber	Yes
Egan	Yes
Muegge	No; 1 violation: XLOGP3>5
Bioavailability Score	0.55
**Medicinal Chemistry**	
PAINS	0 alert
Brenk	0 alert
Leadlikeness	No; 2 violations: MW>350, XLOGP3>3.5
Synthetic accessibility	3.99

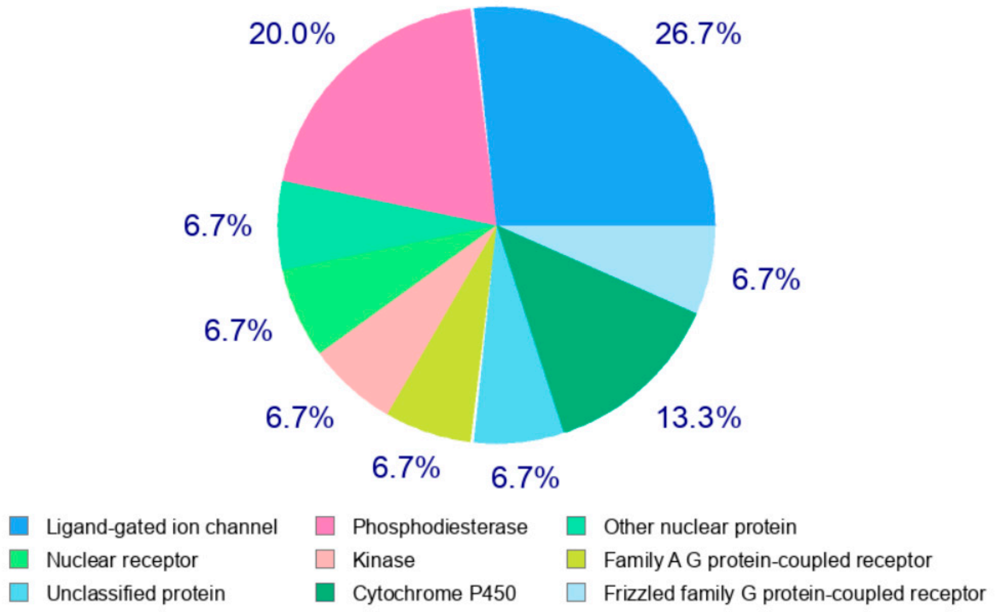

ADME analysis results and probability of molecular targets of compound **1c**

Molecule 1

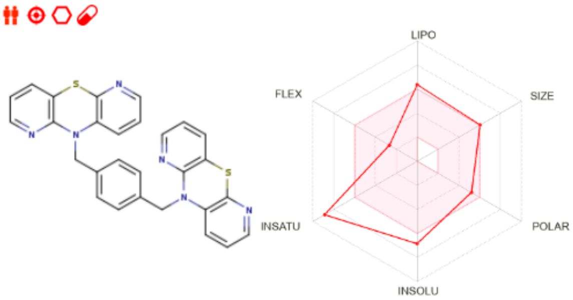

SMILES c1cnc2c(c1)N(Cc1ccc(cc1)CN1c3ncccc3Sc3c1cccn3)c1c(S2)cccn1

Physicochemical Properties	
Formula	C28H20N6S2
Molecular weight	504.63 g/mol
Num. heavy atoms	36
Num. arom. heavy atoms	30
Fraction Csp3	0.07
Num. rotatable bonds	4
Num. H-bond acceptors	4
Num. H-bond donors	0
Molar Refractivity	149.60
TPSA	108.64 Å²

Lipophilicity	
Log $P_{o/w}$ (iLOGP)	3.76
Log $P_{o/w}$ (XLOGP3)	5.62
Log $P_{o/w}$ (WLOGP)	5.81
Log $P_{o/w}$ (MLOGP)	4.07
Log $P_{o/w}$ (SILICOS-IT)	4.67
Consensus Log $P_{o/w}$	4.78

Water Solubility	
Log *S* (ESOL)	-6.86
Solubility	6.93e-05 mg/ml ; 1.37e-07 mol/l
Class	Poorly soluble
Log *S* (Ali)	-7.66
Solubility	1.09e-05 mg/ml ; 2.17e-08 mol/l
Class	Poorly soluble
Log *S* (SILICOS-IT)	-10.48
Solubility	1.67e-08 mg/ml ; 3.30e-11 mol/l
Class	Insoluble

Pharmacokinetics	
GI absorption	Low
BBB permeant	No
P-gp substrate	Yes
CYP1A2 inhibitor	No
CYP2C19 inhibitor	Yes
CYP2C9 inhibitor	Yes
CYP2D6 inhibitor	No
CYP3A4 inhibitor	Yes
Log K_p (skin permeation)	-5.39 cm/s

Druglikeness	
Lipinski	Yes; 1 violation: MW>500
Ghose	No; 3 violations: MW>480, WLOGP>5.6, MR>130
Veber	Yes
Egan	Yes
Muegge	No; 1 violation: XLOGP3>5
Bioavailability Score	0.55

Medicinal Chemistry	
PAINS	0 alert
Brenk	0 alert
Leadlikeness	No; 2 violations: MW>350, XLOGP3>3.5
Synthetic accessibility	3.99

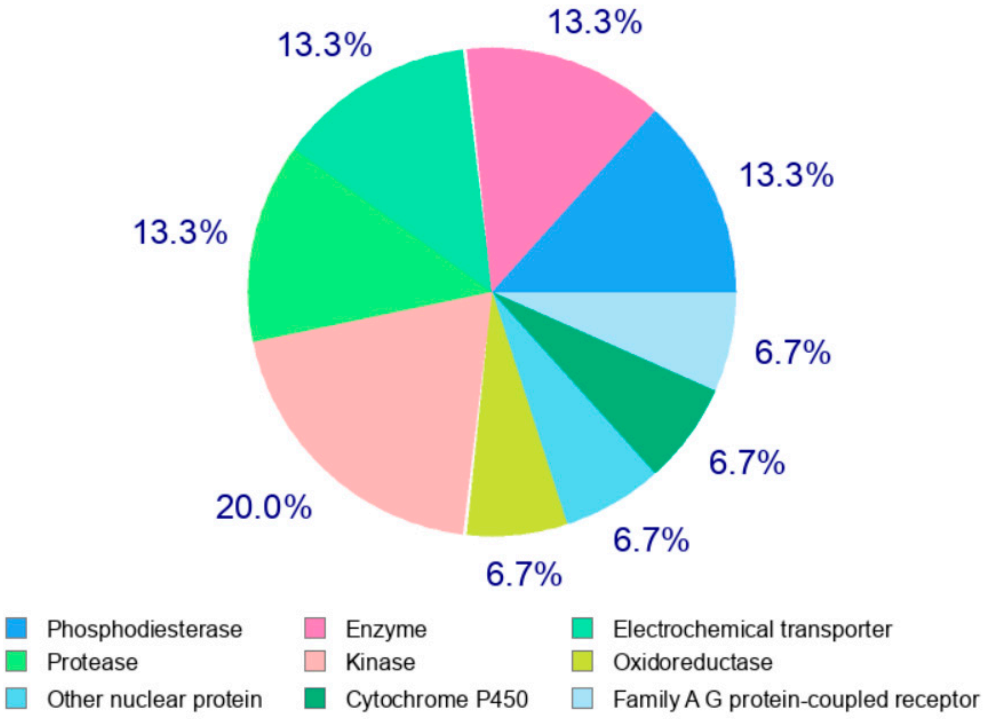

ADME analysis results and probability of molecular targets of compound **1d**

Molecule 1

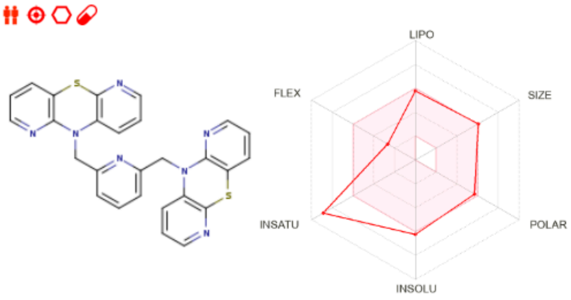

SMILES c1cc(nc(c1)CN1c2ncccc2Sc2c1cccn2)CN1c2ncccc2Sc2c1cccn2

Physicochemical Properties	
Formula	C27H19N7S2
Molecular weight	505.62 g/mol
Num. heavy atoms	36
Num. arom. heavy atoms	30
Fraction Csp3	0.07
Num. rotatable bonds	4
Num. H-bond acceptors	5
Num. H-bond donors	0
Molar Refractivity	147.40
TPSA	121.53 Å²

Lipophilicity	
Log $P_{o/w}$ (iLOGP)	3.22
Log $P_{o/w}$ (XLOGP3)	4.62
Log $P_{o/w}$ (WLOGP)	5.20
Log $P_{o/w}$ (MLOGP)	3.07
Log $P_{o/w}$ (SILICOS-IT)	4.10
Consensus Log $P_{o/w}$	4.04

Water Solubility	
Log *S* (ESOL)	-6.24
Solubility	2.92e-04 mg/ml ; 5.78e-07 mol/l
Class	Poorly soluble
Log *S* (Ali)	-6.90
Solubility	6.40e-05 mg/ml ; 1.27e-07 mol/l
Class	Poorly soluble
Log *S* (SILICOS-IT)	-10.11
Solubility	3.94e-08 mg/ml ; 7.80e-11 mol/l
Class	Insoluble

Pharmacokinetics	
GI absorption	Low
BBB permeant	No
P-gp substrate	Yes
CYP1A2 inhibitor	No
CYP2C19 inhibitor	Yes
CYP2C9 inhibitor	Yes
CYP2D6 inhibitor	No
CYP3A4 inhibitor	Yes
Log K_p (skin permeation)	-6.10 cm/s

Druglikeness	
Lipinski	Yes; 1 violation: MW>500
Ghose	No; 2 violations: MW>480, MR>130
Veber	Yes
Egan	Yes
Muegge	Yes
Bioavailability Score	0.55

Medicinal Chemistry	
PAINS	0 alert
Brenk	0 alert
Leadlikeness	No; 2 violations: MW>350, XLOGP3>3.5
Synthetic accessibility	4.02

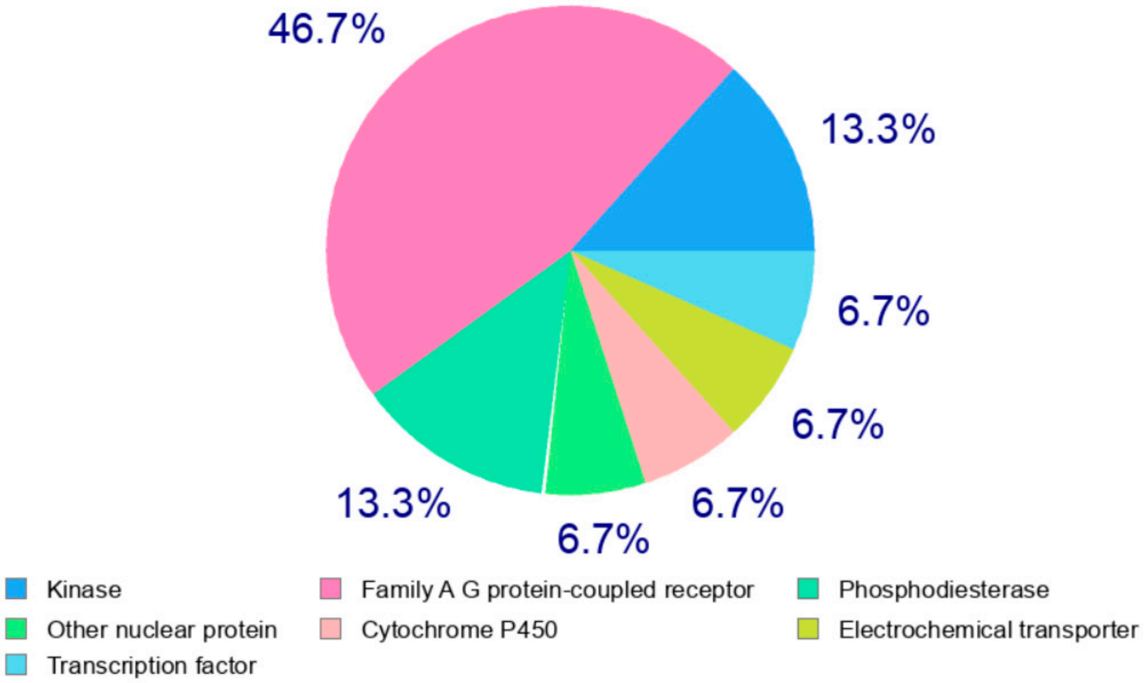

ADME analysis results and probability of molecular targets of compound **2a**

Molecule 1

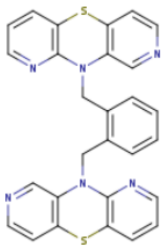

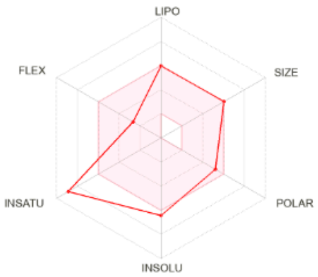

SMILES c1ccc(c(c1)CN1c2cncc2Sc2c1nccc2)CN1c2cncc2Sc2c1nccc2

Physicochemical Properties	
Formula	C28H20N6S2
Molecular weight	504.63 g/mol
Num. heavy atoms	36
Num. arom. heavy atoms	30
Fraction Csp3	0.07
Num. rotatable bonds	4
Num. H-bond acceptors	4
Num. H-bond donors	0
Molar Refractivity	149.60
TPSA	108.64 Å²

Lipophilicity	
Log $P_{o/w}$ (iLOGP)	3.62
Log $P_{o/w}$ (XLOGP3)	4.95
Log $P_{o/w}$ (WLOGP)	5.81
Log $P_{o/w}$ (MLOGP)	4.07
Log $P_{o/w}$ (SILICOS-IT)	4.67
Consensus Log $P_{o/w}$	4.62

Water Solubility	
Log *S* (ESOL)	-6.44
Solubility	1.83e-04 mg/ml ; 3.63e-07 mol/l
Class	Poorly soluble
Log *S* (Ali)	-6.97
Solubility	5.42e-05 mg/ml ; 1.07e-07 mol/l
Class	Poorly soluble
Log *S* (SILICOS-IT)	-10.48
Solubility	1.67e-08 mg/ml ; 3.30e-11 mol/l
Class	Insoluble

Pharmacokinetics	
GI absorption	Low
BBB permeant	No
P-gp substrate	Yes
CYP1A2 inhibitor	No
CYP2C19 inhibitor	Yes
CYP2C9 inhibitor	Yes
CYP2D6 inhibitor	No
CYP3A4 inhibitor	Yes
Log K_p (skin permeation)	-5.86 cm/s

Druglikeness	
Lipinski	Yes; 1 violation: MW>500
Ghose	No; 3 violations: MW>480, WLOGP>5.6, MR>130
Veber	Yes
Egan	Yes
Muegge	Yes
Bioavailability Score	0.55

Medicinal Chemistry	
PAINS	0 alert
Brenk	0 alert
Leadlikeness	No; 2 violations: MW>350, XLOGP3>3.5
Synthetic accessibility	3.93

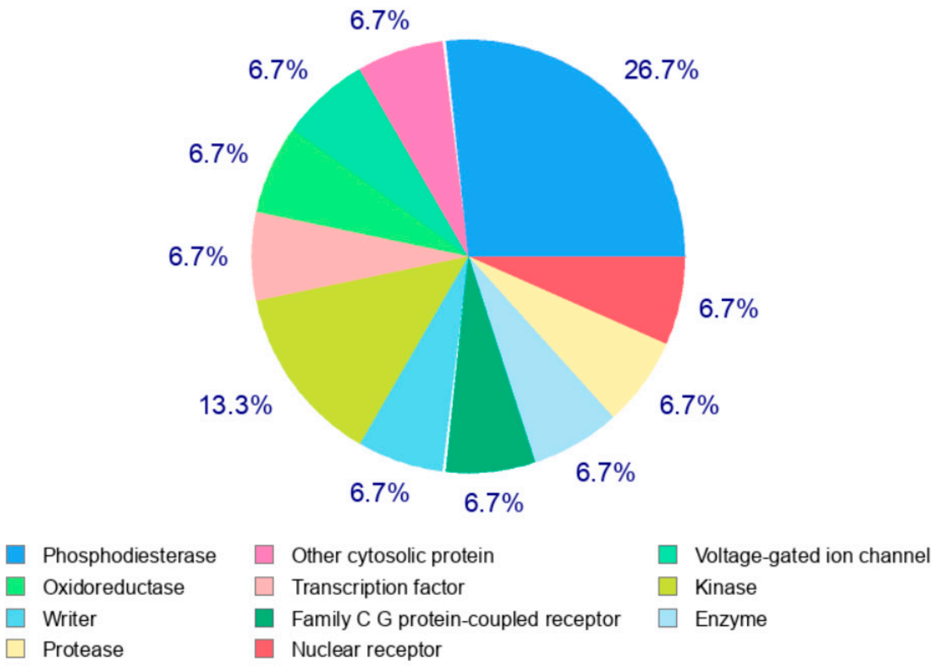

ADME analysis results and probability of molecular targets of compound **2b**

Molecule 1

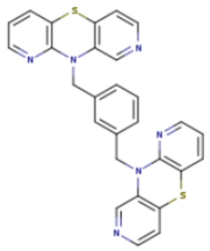

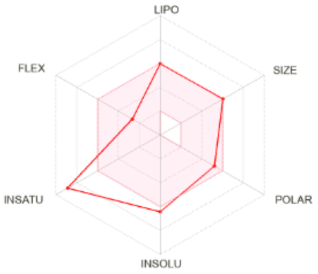

SMILES c1cc(cc(c1)CN1c2cncc2Sc2c1nccc2)CN1c2cncc2Sc2c1nccc2

Physicochemical Properties	
Formula	C28H20N6S2
Molecular weight	504.63 g/mol
Num. heavy atoms	36
Num. arom. heavy atoms	30
Fraction Csp3	0.07
Num. rotatable bonds	4
Num. H-bond acceptors	4
Num. H-bond donors	0
Molar Refractivity	149.60
TPSA	108.64 Å²

Lipophilicity	
Log $P_{o/w}$ (iLOGP)	3.34
Log $P_{o/w}$ (XLOGP3)	4.95
Log $P_{o/w}$ (WLOGP)	5.81
Log $P_{o/w}$ (MLOGP)	4.07
Log $P_{o/w}$ (SILICOS-IT)	4.67
Consensus Log $P_{o/w}$	4.57

Water Solubility	
Log *S* (ESOL)	-6.44
Solubility	1.83e-04 mg/ml ; 3.63e-07 mol/l
Class	Poorly soluble
Log *S* (Ali)	-6.97
Solubility	5.42e-05 mg/ml ; 1.07e-07 mol/l
Class	Poorly soluble
Log *S* (SILICOS-IT)	-10.48
Solubility	1.67e-08 mg/ml ; 3.30e-11 mol/l
Class	Insoluble

Pharmacokinetics	
GI absorption	Low
BBB permeant	No
P-gp substrate	Yes
CYP1A2 inhibitor	No
CYP2C19 inhibitor	Yes
CYP2C9 inhibitor	Yes
CYP2D6 inhibitor	No
CYP3A4 inhibitor	Yes
Log K_p (skin permeation)	-5.86 cm/s

Druglikeness	
Lipinski	Yes; 1 violation: MW>500
Ghose	No; 3 violations: MW>480, WLOGP>5.6, MR>130
Veber	Yes
Egan	Yes
Muegge	Yes
Bioavailability Score	0.55

Medicinal Chemistry	
PAINS	0 alert
Brenk	0 alert
Leadlikeness	No; 2 violations: MW>350, XLOGP3>3.5
Synthetic accessibility	3.94

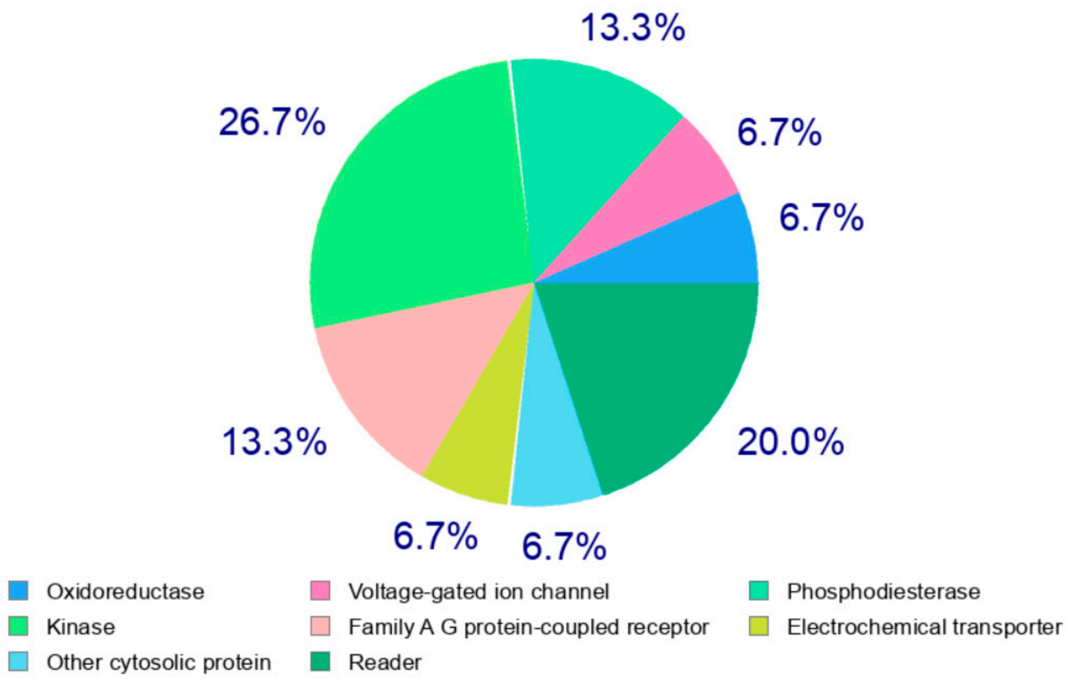

ADME analysis results and probability of molecular targets of compound **2c**

Molecule 1

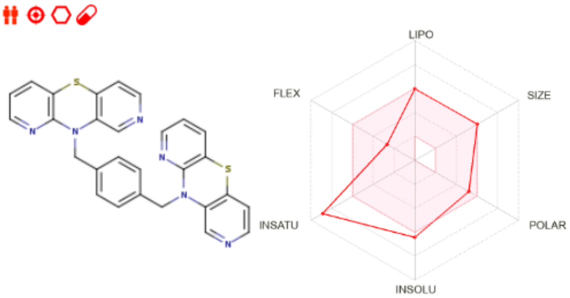

SMILES c1cnc2c(c1)Sc1c(N2Cc2ccc(cc2)CN2c3cncc3Sc3c2nccc3)cnc1

Physicochemical Properties	
Formula	C28H20N6S2
Molecular weight	504.63 g/mol
Num. heavy atoms	36
Num. arom. heavy atoms	30
Fraction Csp3	0.07
Num. rotatable bonds	4
Num. H-bond acceptors	4
Num. H-bond donors	0
Molar Refractivity	149.60
TPSA	108.64 Å²

Lipophilicity	
Log $P_{o/w}$ (iLOGP)	3.85
Log $P_{o/w}$ (XLOGP3)	4.95
Log $P_{o/w}$ (WLOGP)	5.81
Log $P_{o/w}$ (MLOGP)	4.07
Log $P_{o/w}$ (SILICOS-IT)	4.67
Consensus Log $P_{o/w}$	4.67

Water Solubility	
Log *S* (ESOL)	-6.44
Solubility	1.83e-04 mg/ml ; 3.63e-07 mol/l
Class	Poorly soluble
Log *S* (Ali)	-6.97
Solubility	5.42e-05 mg/ml ; 1.07e-07 mol/l
Class	Poorly soluble
Log *S* (SILICOS-IT)	-10.48
Solubility	1.67e-08 mg/ml ; 3.30e-11 mol/l
Class	Insoluble

Pharmacokinetics	
GI absorption	Low
BBB permeant	No
P-gp substrate	Yes
CYP1A2 inhibitor	No
CYP2C19 inhibitor	Yes
CYP2C9 inhibitor	Yes
CYP2D6 inhibitor	No
CYP3A4 inhibitor	Yes
Log K_p (skin permeation)	-5.86 cm/s

Druglikeness	
Lipinski	Yes; 1 violation: MW>500
Ghose	No; 3 violations: MW>480, WLOGP>5.6, MR>130
Veber	Yes
Egan	Yes
Muegge	Yes
Bioavailability Score	0.55

Medicinal Chemistry	
PAINS	0 alert
Brenk	0 alert
Leadlikeness	No; 2 violations: MW>350, XLOGP3>3.5
Synthetic accessibility	3.94

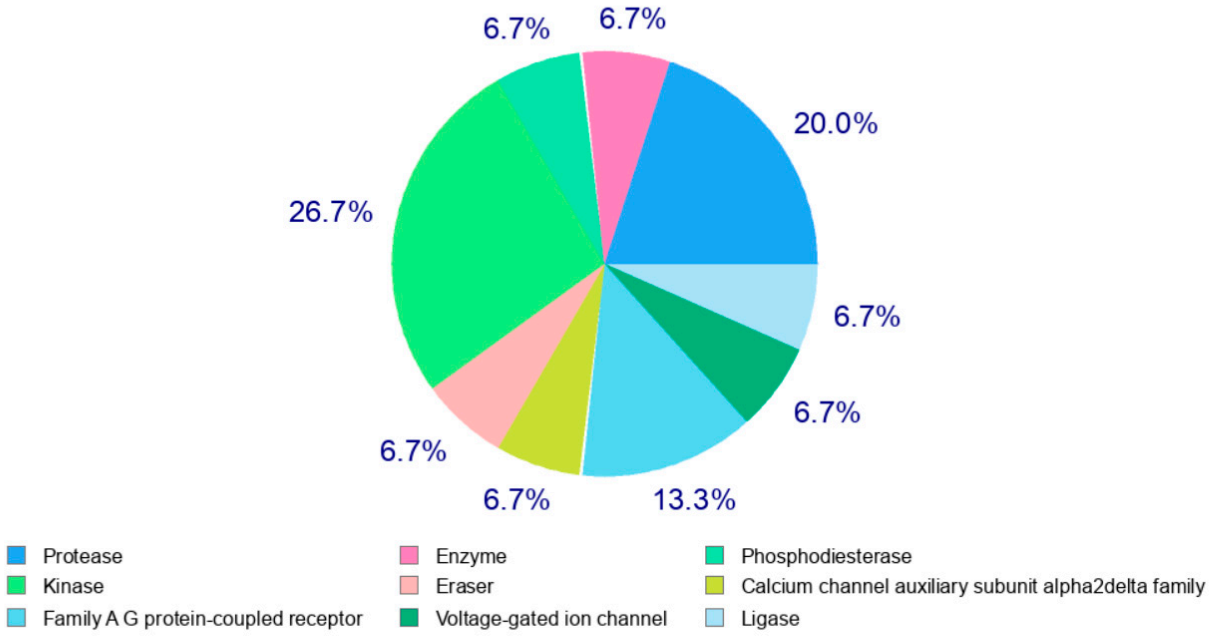

ADME analysis results and probability of molecular targets of compound **2d**

Molecule 1

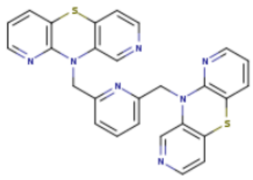

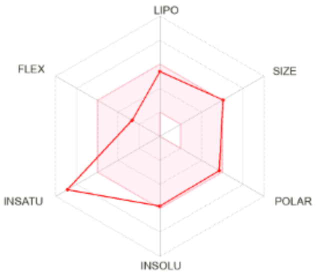

SMILES c1cc(nc(c1)CN1c2cnccc2Sc2c1nccc2)CN1c2cnccc2Sc2c1nccc2

Physicochemical Properties	
Formula	C27H19N7S2
Molecular weight	505.62 g/mol
Num. heavy atoms	36
Num. arom. heavy atoms	30
Fraction Csp3	0.07
Num. rotatable bonds	4
Num. H-bond acceptors	5
Num. H-bond donors	0
Molar Refractivity	147.40
TPSA	121.53 Å²

Lipophilicity	
Log $P_{o/w}$ (iLOGP)	3.67
Log $P_{o/w}$ (XLOGP3)	3.95
Log $P_{o/w}$ (WLOGP)	5.20
Log $P_{o/w}$ (MLOGP)	3.07
Log $P_{o/w}$ (SILICOS-IT)	4.10
Consensus Log $P_{o/w}$	4.00

Water Solubility	
Log *S* (ESOL)	-5.82
Solubility	7.72e-04 mg/ml ; 1.53e-06 mol/l
Class	Moderately soluble
Log *S* (Ali)	-6.20
Solubility	3.17e-04 mg/ml ; 6.28e-07 mol/l
Class	Poorly soluble
Log *S* (SILICOS-IT)	-10.11
Solubility	3.94e-08 mg/ml ; 7.80e-11 mol/l
Class	Insoluble

Pharmacokinetics	
GI absorption	Low
BBB permeant	No
P-gp substrate	Yes
CYP1A2 inhibitor	No
CYP2C19 inhibitor	Yes
CYP2C9 inhibitor	Yes
CYP2D6 inhibitor	No
CYP3A4 inhibitor	Yes
Log K_p (skin permeation)	-6.58 cm/s

Druglikeness	
Lipinski	Yes; 1 violation: MW>500
Ghose	No; 2 violations: MW>480, MR>130
Veber	Yes
Egan	Yes
Muegge	Yes
Bioavailability Score	0.55

Medicinal Chemistry	
PAINS	0 alert
Brenk	0 alert
Leadlikeness	No; 2 violations: MW>350, XLOGP3>3.5
Synthetic accessibility	3.98

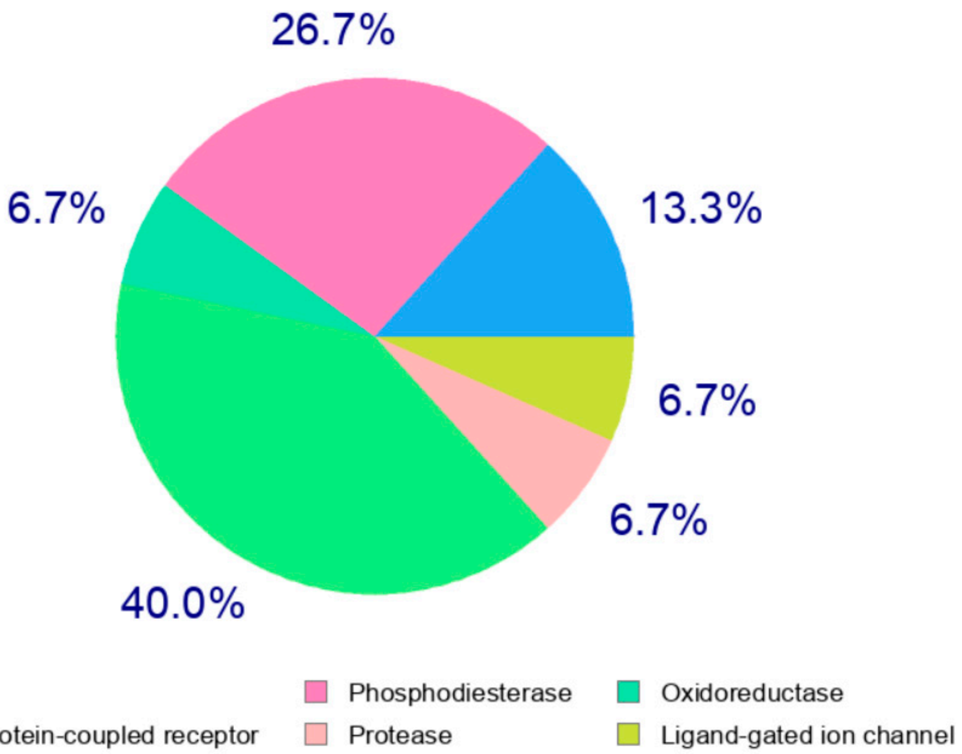

ADME analysis results and probability of molecular targets of compound **3a**

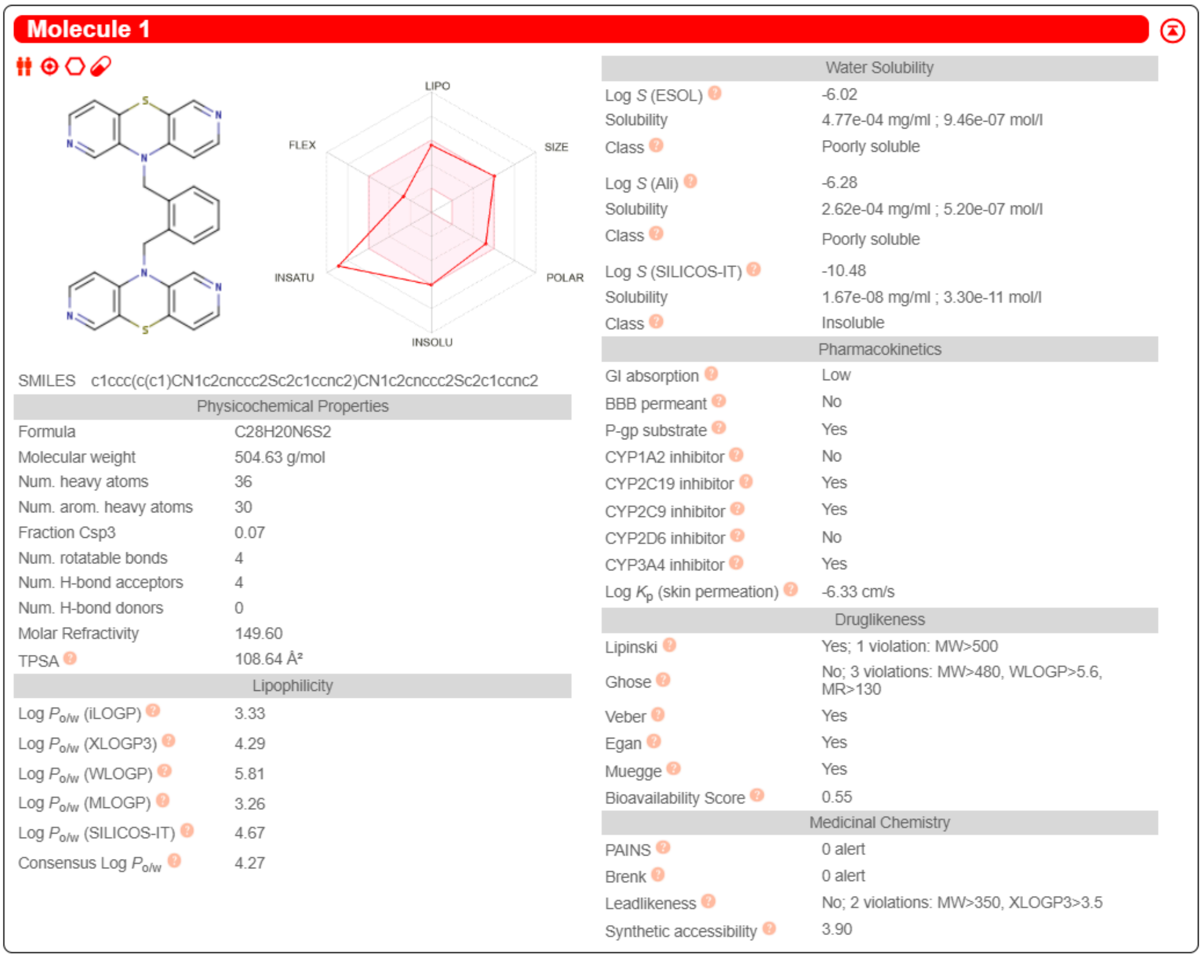

Molecule 1

SMILES c1ccc(c(c1)CN1c2cnccc2Sc2c1ccnc2)CN1c2cnccc2Sc2c1ccnc2

Physicochemical Properties	
Formula	C28H20N6S2
Molecular weight	504.63 g/mol
Num. heavy atoms	36
Num. arom. heavy atoms	30
Fraction Csp3	0.07
Num. rotatable bonds	4
Num. H-bond acceptors	4
Num. H-bond donors	0
Molar Refractivity	149.60
TPSA	108.64 Å²

Lipophilicity	
Log $P_{o/w}$ (iLOGP)	3.33
Log $P_{o/w}$ (XLOGP3)	4.29
Log $P_{o/w}$ (WLOGP)	5.81
Log $P_{o/w}$ (MLOGP)	3.26
Log $P_{o/w}$ (SILICOS-IT)	4.67
Consensus Log $P_{o/w}$	4.27

Water Solubility	
Log *S* (ESOL)	-6.02
Solubility	4.77e-04 mg/ml ; 9.46e-07 mol/l
Class	Poorly soluble
Log *S* (Ali)	-6.28
Solubility	2.62e-04 mg/ml ; 5.20e-07 mol/l
Class	Poorly soluble
Log *S* (SILICOS-IT)	-10.48
Solubility	1.67e-08 mg/ml ; 3.30e-11 mol/l
Class	Insoluble

Pharmacokinetics	
GI absorption	Low
BBB permeant	No
P-gp substrate	Yes
CYP1A2 inhibitor	No
CYP2C19 inhibitor	Yes
CYP2C9 inhibitor	Yes
CYP2D6 inhibitor	No
CYP3A4 inhibitor	Yes
Log K_p (skin permeation)	-6.33 cm/s

Druglikeness	
Lipinski	Yes; 1 violation: MW>500
Ghose	No; 3 violations: MW>480, WLOGP>5.6, MR>130
Veber	Yes
Egan	Yes
Muegge	Yes
Bioavailability Score	0.55

Medicinal Chemistry	
PAINS	0 alert
Brenk	0 alert
Leadlikeness	No; 2 violations: MW>350, XLOGP3>3.5
Synthetic accessibility	3.90

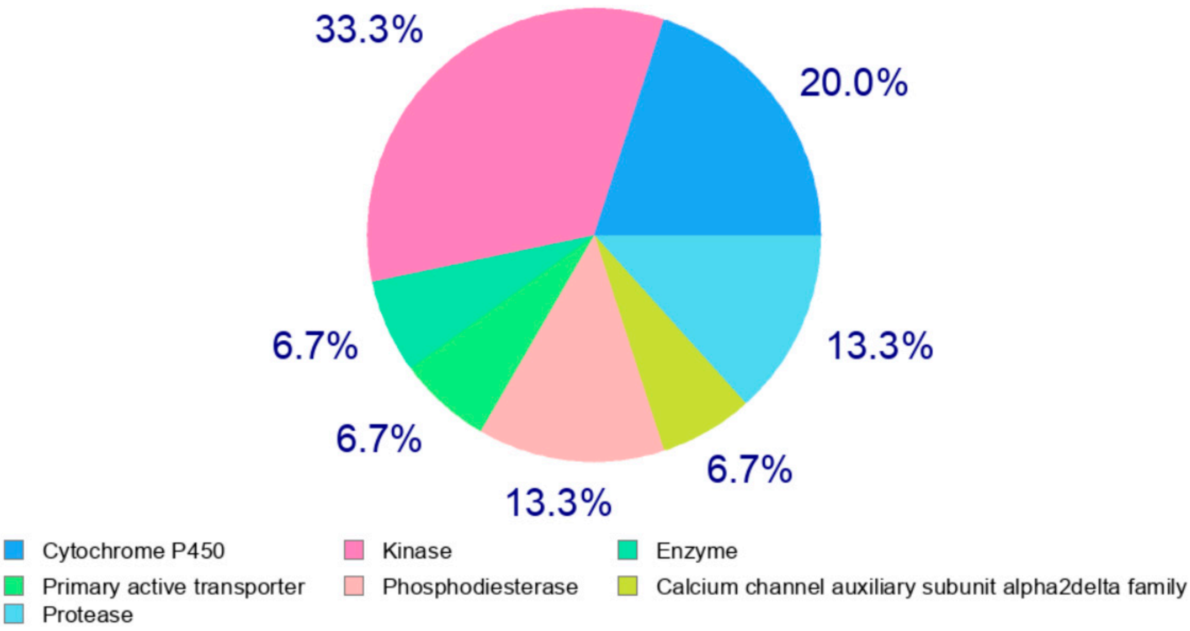

ADME analysis results and probability of molecular targets of compound **3b**

Molecule 1

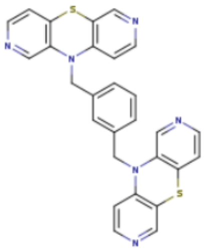

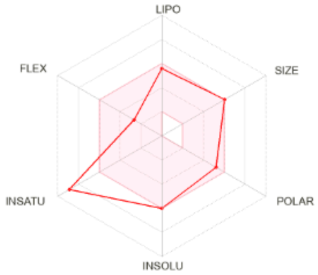

SMILES c1cc(cc(c1)CN1c2cnccc2Sc2c1ccnc2)CN1c2cnccc2Sc2c1ccnc2

Physicochemical Properties	
Formula	C28H20N6S2
Molecular weight	504.63 g/mol
Num. heavy atoms	36
Num. arom. heavy atoms	30
Fraction Csp3	0.07
Num. rotatable bonds	4
Num. H-bond acceptors	4
Num. H-bond donors	0
Molar Refractivity	149.60
TPSA	108.64 Å²

Lipophilicity	
Log $P_{o/w}$ (iLOGP)	3.70
Log $P_{o/w}$ (XLOGP3)	4.29
Log $P_{o/w}$ (WLOGP)	5.81
Log $P_{o/w}$ (MLOGP)	3.26
Log $P_{o/w}$ (SILICOS-IT)	4.67
Consensus Log $P_{o/w}$	4.35

Water Solubility	
Log *S* (ESOL)	-6.02
Solubility	4.77e-04 mg/ml ; 9.46e-07 mol/l
Class	Poorly soluble
Log *S* (Ali)	-6.28
Solubility	2.62e-04 mg/ml ; 5.20e-07 mol/l
Class	Poorly soluble
Log *S* (SILICOS-IT)	-10.48
Solubility	1.67e-08 mg/ml ; 3.30e-11 mol/l
Class	Insoluble

Pharmacokinetics	
GI absorption	Low
BBB permeant	No
P-gp substrate	Yes
CYP1A2 inhibitor	No
CYP2C19 inhibitor	Yes
CYP2C9 inhibitor	Yes
CYP2D6 inhibitor	No
CYP3A4 inhibitor	Yes
Log K_p (skin permeation)	-6.33 cm/s

Druglikeness	
Lipinski	Yes; 1 violation: MW>500
Ghose	No; 3 violations: MW>480, WLOGP>5.6, MR>130
Veber	Yes
Egan	Yes
Muegge	Yes
Bioavailability Score	0.55

Medicinal Chemistry	
PAINS	0 alert
Brenk	0 alert
Leadlikeness	No; 2 violations: MW>350, XLOGP3>3.5
Synthetic accessibility	3.90

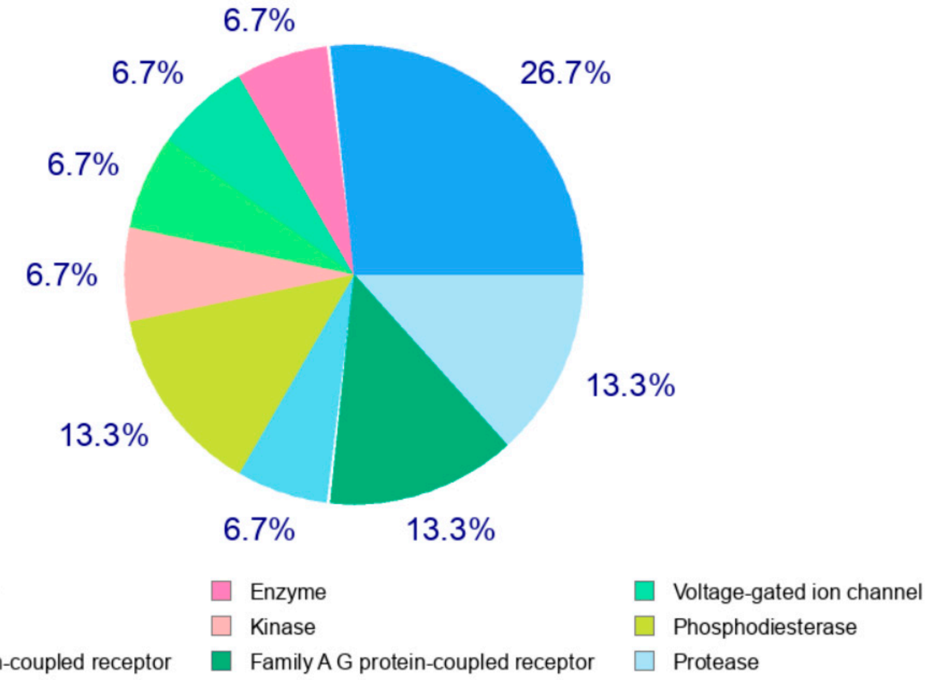

ADME analysis results and probability of molecular targets of compound **3c**

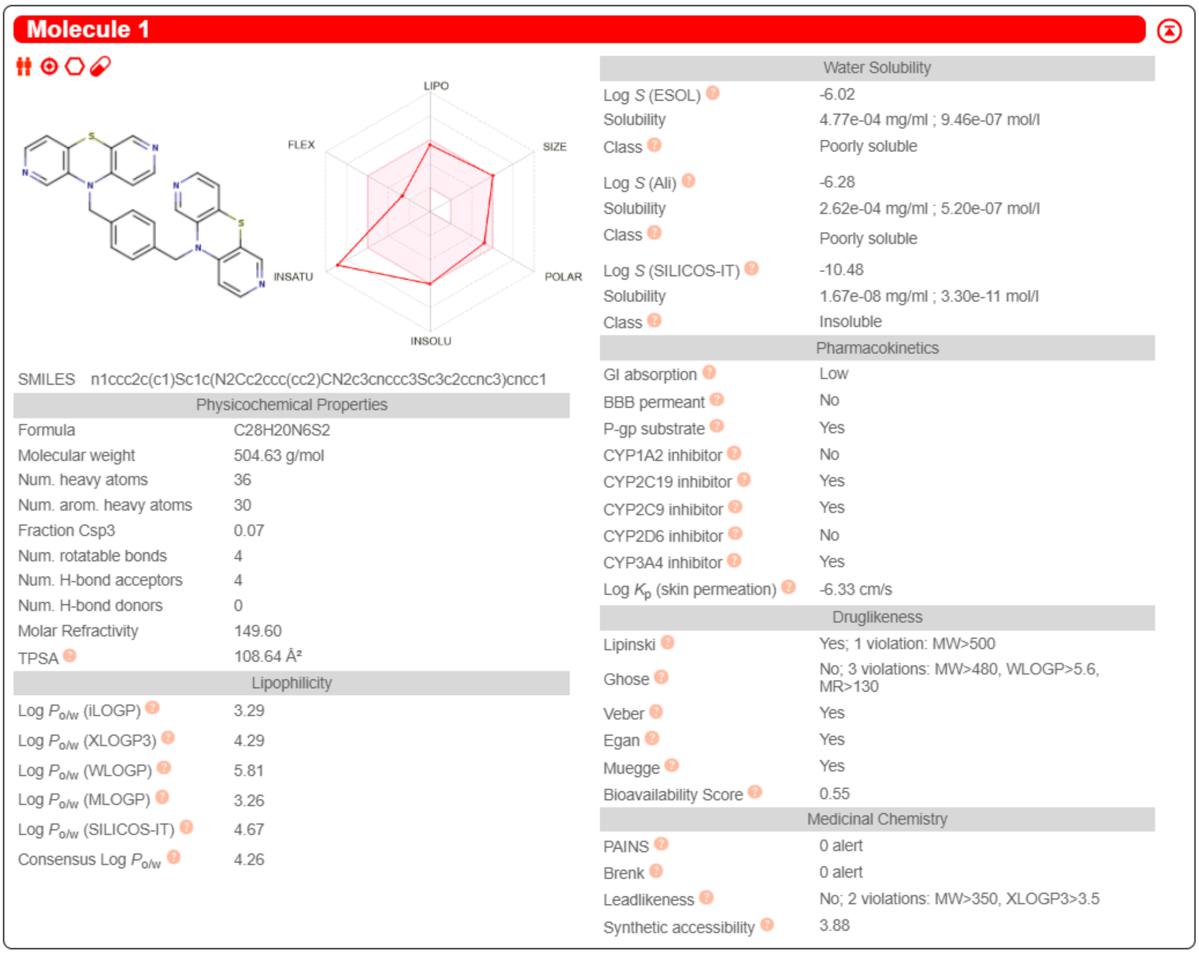

Molecule 1

SMILES n1ccc2c(c1)Sc1c(N2Cc2ccc(cc2)CN2c3cnccc3Sc3c2ccnc3)cnc1

Physicochemical Properties	
Formula	C28H20N6S2
Molecular weight	504.63 g/mol
Num. heavy atoms	36
Num. arom. heavy atoms	30
Fraction Csp3	0.07
Num. rotatable bonds	4
Num. H-bond acceptors	4
Num. H-bond donors	0
Molar Refractivity	149.60
TPSA	108.64 Å²

Lipophilicity	
Log $P_{o/w}$ (iLOGP)	3.29
Log $P_{o/w}$ (XLOGP3)	4.29
Log $P_{o/w}$ (WLOGP)	5.81
Log $P_{o/w}$ (MLOGP)	3.26
Log $P_{o/w}$ (SILICOS-IT)	4.67
Consensus Log $P_{o/w}$	4.26

Water Solubility	
Log *S* (ESOL)	-6.02
Solubility	4.77e-04 mg/ml ; 9.46e-07 mol/l
Class	Poorly soluble
Log *S* (Ali)	-6.28
Solubility	2.62e-04 mg/ml ; 5.20e-07 mol/l
Class	Poorly soluble
Log *S* (SILICOS-IT)	-10.48
Solubility	1.67e-08 mg/ml ; 3.30e-11 mol/l
Class	Insoluble

Pharmacokinetics	
GI absorption	Low
BBB permeant	No
P-gp substrate	Yes
CYP1A2 inhibitor	No
CYP2C19 inhibitor	Yes
CYP2C9 inhibitor	Yes
CYP2D6 inhibitor	No
CYP3A4 inhibitor	Yes
Log K_p (skin permeation)	-6.33 cm/s

Druglikeness	
Lipinski	Yes; 1 violation: MW>500
Ghose	No; 3 violations: MW>480, WLOGP>5.6, MR>130
Veber	Yes
Egan	Yes
Muegge	Yes
Bioavailability Score	0.55

Medicinal Chemistry	
PAINS	0 alert
Brenk	0 alert
Leadlikeness	No; 2 violations: MW>350, XLOGP3>3.5
Synthetic accessibility	3.88

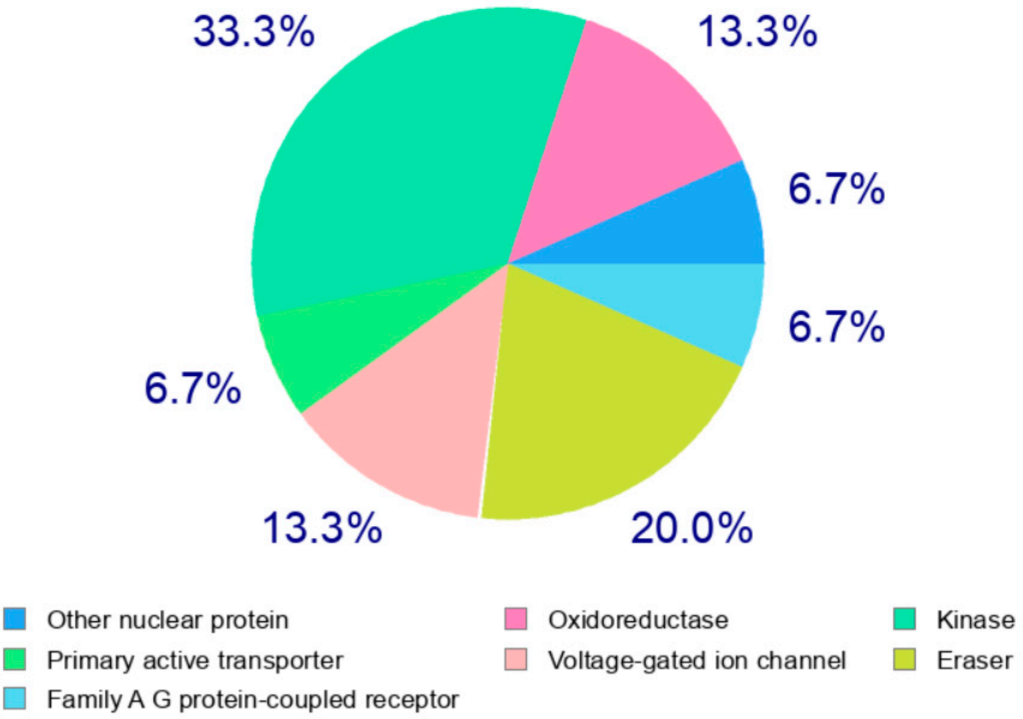

ADME analysis results and probability of molecular targets of compound **3d**

Molecule 1

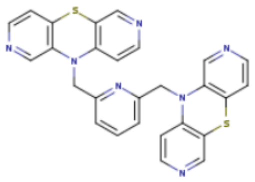

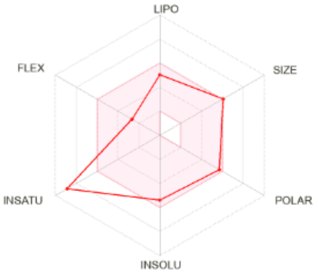

SMILES c1cc(nc(c1)CN1c2cnccc2Sc2c1ccnc2)CN1c2cnccc2Sc2c1ccnc2

Physicochemical Properties	
Formula	C27H19N7S2
Molecular weight	505.62 g/mol
Num. heavy atoms	36
Num. arom. heavy atoms	30
Fraction Csp3	0.07
Num. rotatable bonds	4
Num. H-bond acceptors	5
Num. H-bond donors	0
Molar Refractivity	147.40
TPSA	121.53 Å²

Lipophilicity	
Log $P_{o/w}$ (iLOGP)	3.30
Log $P_{o/w}$ (XLOGP3)	3.29
Log $P_{o/w}$ (WLOGP)	5.20
Log $P_{o/w}$ (MLOGP)	2.26
Log $P_{o/w}$ (SILICOS-IT)	4.10
Consensus Log $P_{o/w}$	3.63

Water Solubility	
Log *S* (ESOL)	-5.40
Solubility	2.01e-03 mg/ml ; 3.98e-06 mol/l
Class	Moderately soluble
Log *S* (Ali)	-5.52
Solubility	1.54e-03 mg/ml ; 3.04e-06 mol/l
Class	Moderately soluble
Log *S* (SILICOS-IT)	-10.11
Solubility	3.94e-08 mg/ml ; 7.80e-11 mol/l
Class	Insoluble

Pharmacokinetics	
GI absorption	Low
BBB permeant	No
P-gp substrate	Yes
CYP1A2 inhibitor	No
CYP2C19 inhibitor	Yes
CYP2C9 inhibitor	Yes
CYP2D6 inhibitor	Yes
CYP3A4 inhibitor	Yes
Log K_p (skin permeation)	-7.05 cm/s

Druglikeness	
Lipinski	Yes; 1 violation: MW>500
Ghose	No; 2 violations: MW>480, MR>130
Veber	Yes
Egan	Yes
Muegge	Yes
Bioavailability Score	0.55

Medicinal Chemistry	
PAINS	0 alert
Brenk	0 alert
Leadlikeness	No; 1 violation: MW>350
Synthetic accessibility	3.97

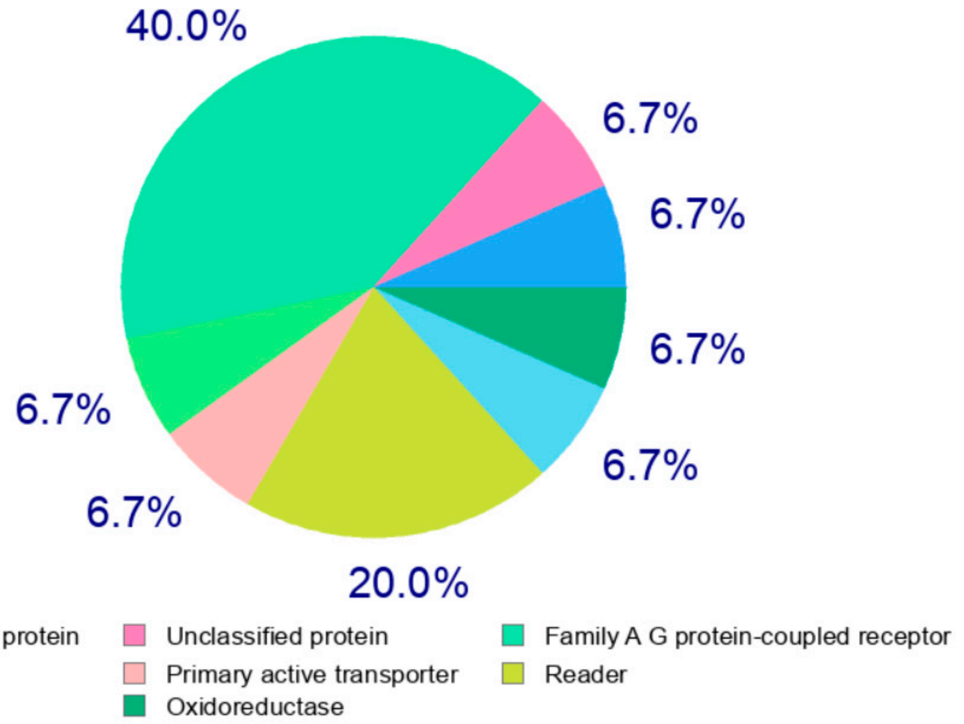

ADME analysis results and probability of molecular targets of compound **4a**

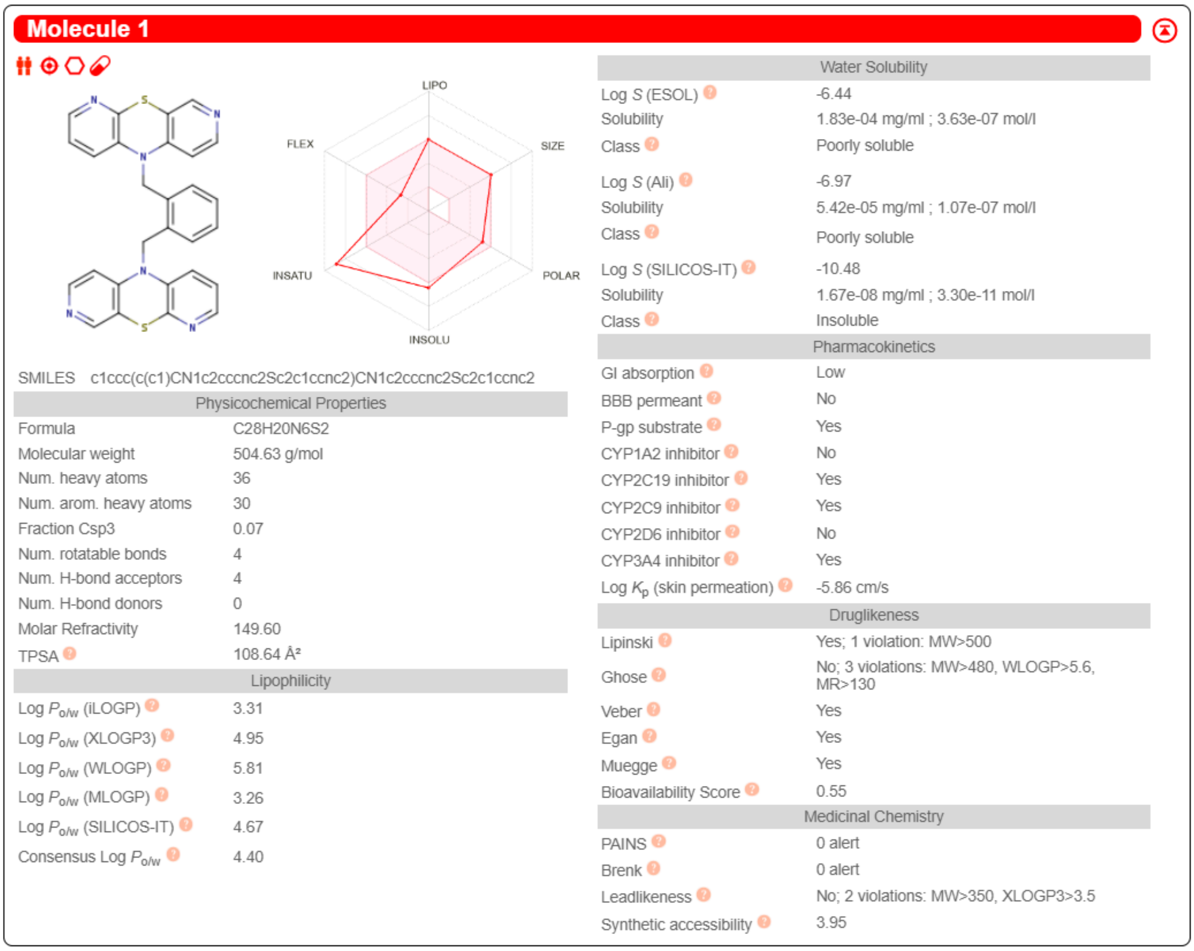

SMILES c1ccc(c(c1)CN1c2cccnc2Sc2c1ccnc2)CN1c2cccnc2Sc2c1ccnc2

Physicochemical Properties	
Formula	C28H20N6S2
Molecular weight	504.63 g/mol
Num. heavy atoms	36
Num. arom. heavy atoms	30
Fraction Csp3	0.07
Num. rotatable bonds	4
Num. H-bond acceptors	4
Num. H-bond donors	0
Molar Refractivity	149.60
TPSA	108.64 Å²

Lipophilicity	
Log $P_{o/w}$ (iLOGP)	3.31
Log $P_{o/w}$ (XLOGP3)	4.95
Log $P_{o/w}$ (WLOGP)	5.81
Log $P_{o/w}$ (MLOGP)	3.26
Log $P_{o/w}$ (SILICOS-IT)	4.67
Consensus Log $P_{o/w}$	4.40

Water Solubility	
Log *S* (ESOL)	-6.44
Solubility	1.83e-04 mg/ml ; 3.63e-07 mol/l
Class	Poorly soluble
Log *S* (Ali)	-6.97
Solubility	5.42e-05 mg/ml ; 1.07e-07 mol/l
Class	Poorly soluble
Log *S* (SILICOS-IT)	-10.48
Solubility	1.67e-08 mg/ml ; 3.30e-11 mol/l
Class	Insoluble

Pharmacokinetics	
GI absorption	Low
BBB permeant	No
P-gp substrate	Yes
CYP1A2 inhibitor	No
CYP2C19 inhibitor	Yes
CYP2C9 inhibitor	Yes
CYP2D6 inhibitor	No
CYP3A4 inhibitor	Yes
Log K_p (skin permeation)	-5.86 cm/s

Druglikeness	
Lipinski	Yes; 1 violation: MW>500
Ghose	No; 3 violations: MW>480, WLOGP>5.6, MR>130
Veber	Yes
Egan	Yes
Muegge	Yes
Bioavailability Score	0.55

Medicinal Chemistry	
PAINS	0 alert
Brenk	0 alert
Leadlikeness	No; 2 violations: MW>350, XLOGP3>3.5
Synthetic accessibility	3.95

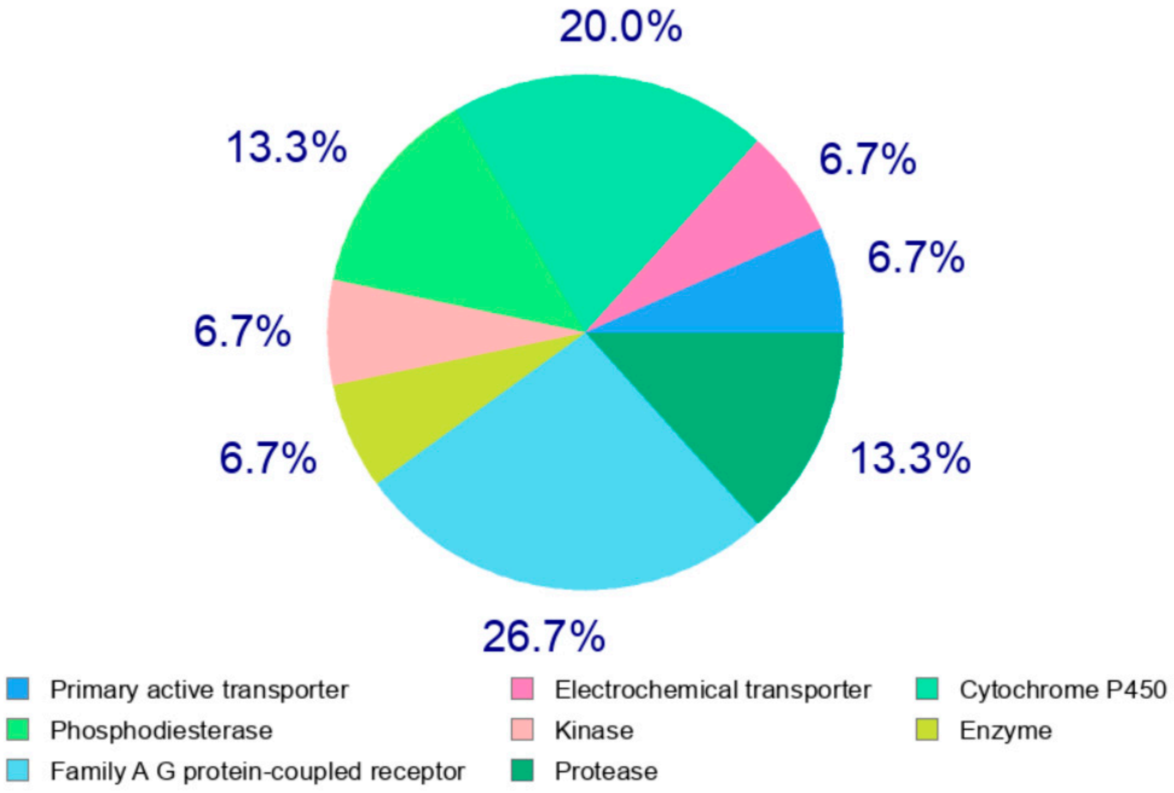

ADME analysis results and probability of molecular targets of compound **4b**

Molecule 1

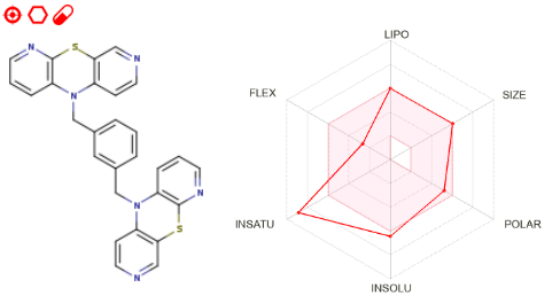

SMILES c1cc(cc(c1)CN1c2cccnc2Sc2c1ccnc2)CN1c2cccnc2Sc2c1ccnc2

Physicochemical Properties	
Formula	C28H20N6S2
Molecular weight	504.63 g/mol
Num. heavy atoms	36
Num. arom. heavy atoms	30
Fraction Csp3	0.07
Num. rotatable bonds	4
Num. H-bond acceptors	4
Num. H-bond donors	0
Molar Refractivity	149.60
TPSA	108.64 Å²

Lipophilicity	
Log $P_{o/w}$ (iLOGP)	3.28
Log $P_{o/w}$ (XLOGP3)	4.95
Log $P_{o/w}$ (WLOGP)	5.81
Log $P_{o/w}$ (MLOGP)	3.26
Log $P_{o/w}$ (SILICOS-IT)	4.67
Consensus Log $P_{o/w}$	4.39

Water Solubility	
Log *S* (ESOL)	-6.44
Solubility	1.83e-04 mg/ml ; 3.63e-07 mol/l
Class	Poorly soluble
Log *S* (Ali)	-6.97
Solubility	5.42e-05 mg/ml ; 1.07e-07 mol/l
Class	Poorly soluble
Log *S* (SILICOS-IT)	-10.48
Solubility	1.67e-08 mg/ml ; 3.30e-11 mol/l
Class	Insoluble

Pharmacokinetics	
GI absorption	Low
BBB permeant	No
P-gp substrate	Yes
CYP1A2 inhibitor	No
CYP2C19 inhibitor	Yes
CYP2C9 inhibitor	Yes
CYP2D6 inhibitor	No
CYP3A4 inhibitor	Yes
Log K_p (skin permeation)	-5.86 cm/s

Druglikeness	
Lipinski	Yes; 1 violation: MW>500
Ghose	No; 3 violations: MW>480, WLOGP>5.6, MR>130
Veber	Yes
Egan	Yes
Muegge	Yes
Bioavailability Score	0.55

Medicinal Chemistry	
PAINS	0 alert
Brenk	0 alert
Leadlikeness	No; 2 violations: MW>350, XLOGP3>3.5
Synthetic accessibility	3.94

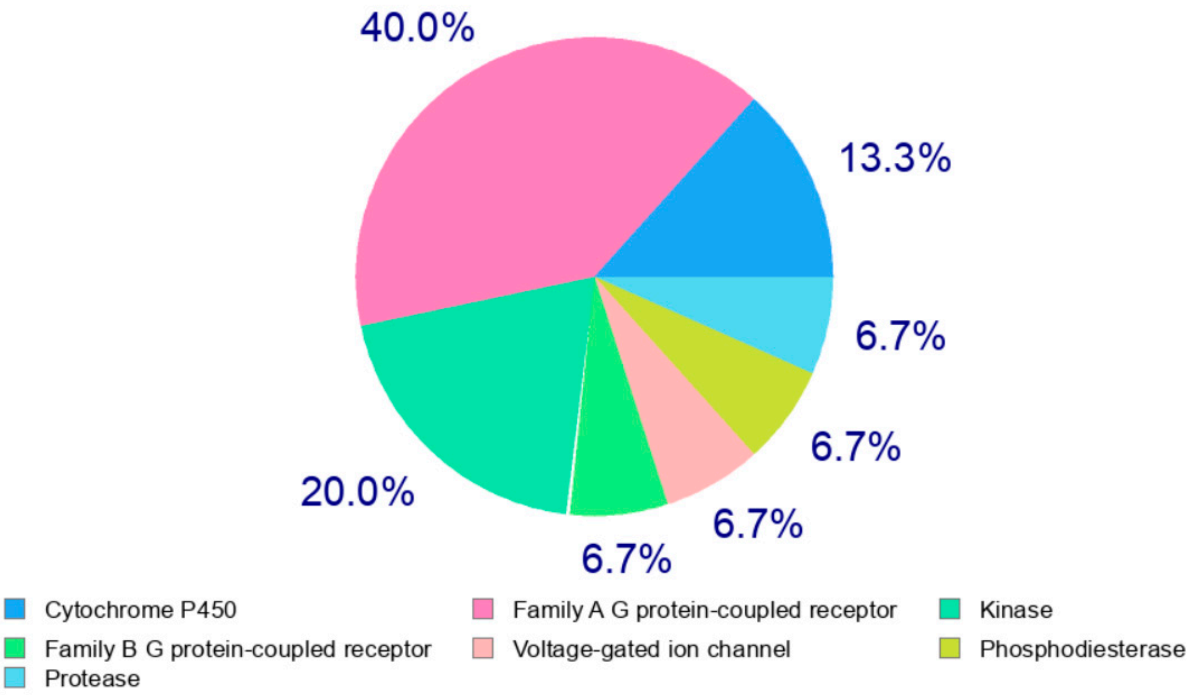

ADME analysis results and probability of molecular targets of compound **4c**

Molecule 1

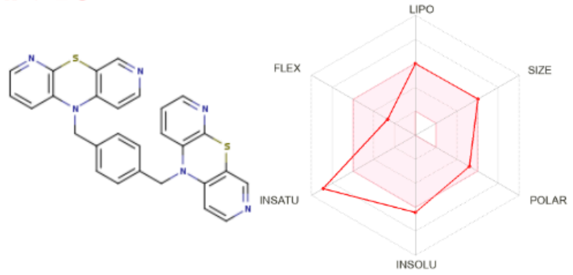

SMILES n1ccc2c(c1)Sc1c(N2Cc2ccc(cc2)CN2c3cccnc3Sc3c2ccnc3)cccn1

Physicochemical Properties	
Formula	C28H20N6S2
Molecular weight	504.63 g/mol
Num. heavy atoms	36
Num. arom. heavy atoms	30
Fraction Csp3	0.07
Num. rotatable bonds	4
Num. H-bond acceptors	4
Num. H-bond donors	0
Molar Refractivity	149.60
TPSA	108.64 Å²

Lipophilicity	
Log $P_{o/w}$ (iLOGP)	3.26
Log $P_{o/w}$ (XLOGP3)	4.95
Log $P_{o/w}$ (WLOGP)	5.81
Log $P_{o/w}$ (MLOGP)	3.26
Log $P_{o/w}$ (SILICOS-IT)	4.67
Consensus Log $P_{o/w}$	4.39

Water Solubility	
Log *S* (ESOL)	-6.44
Solubility	1.83e-04 mg/ml ; 3.63e-07 mol/l
Class	Poorly soluble
Log *S* (Ali)	-6.97
Solubility	5.42e-05 mg/ml ; 1.07e-07 mol/l
Class	Poorly soluble
Log *S* (SILICOS-IT)	-10.48
Solubility	1.67e-08 mg/ml ; 3.30e-11 mol/l
Class	Insoluble

Pharmacokinetics	
GI absorption	Low
BBB permeant	No
P-gp substrate	Yes
CYP1A2 inhibitor	No
CYP2C19 inhibitor	Yes
CYP2C9 inhibitor	Yes
CYP2D6 inhibitor	No
CYP3A4 inhibitor	Yes
Log K_p (skin permeation)	-5.86 cm/s

Druglikeness	
Lipinski	Yes; 1 violation: MW>500
Ghose	No; 3 violations: MW>480, WLOGP>5.6, MR>130
Veber	Yes
Egan	Yes
Muegge	Yes
Bioavailability Score	0.55

Medicinal Chemistry	
PAINS	0 alert
Brenk	0 alert
Leadlikeness	No; 2 violations: MW>350, XLOGP3>3.5
Synthetic accessibility	3.93

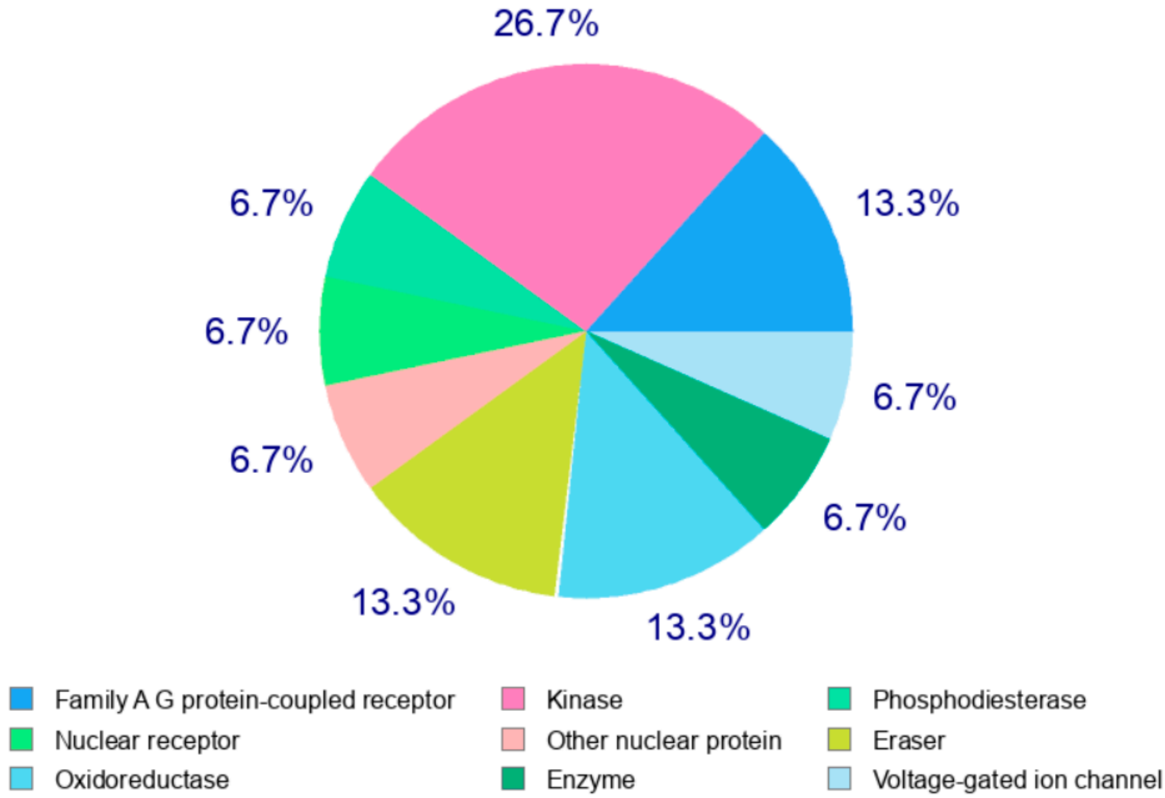

ADME analysis results and probability of molecular targets of compound **4d**

Molecule 1

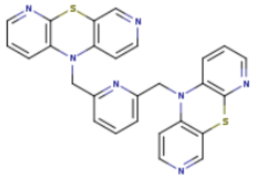

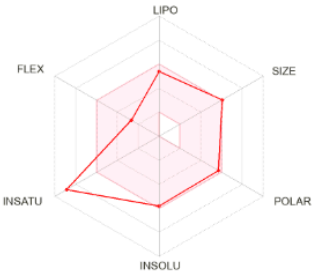

SMILES c1cc(nc(c1)CN1c2cccnc2Sc2c1ccnc2)CN1c2cccnc2Sc2c1ccnc2

Physicochemical Properties	
Formula	C27H19N7S2
Molecular weight	505.62 g/mol
Num. heavy atoms	36
Num. arom. heavy atoms	30
Fraction Csp3	0.07
Num. rotatable bonds	4
Num. H-bond acceptors	5
Num. H-bond donors	0
Molar Refractivity	147.40
TPSA	121.53 Å²

Lipophilicity	
Log $P_{o/w}$ (iLOGP)	3.22
Log $P_{o/w}$ (XLOGP3)	3.95
Log $P_{o/w}$ (WLOGP)	5.20
Log $P_{o/w}$ (MLOGP)	2.26
Log $P_{o/w}$ (SILICOS-IT)	4.10
Consensus Log $P_{o/w}$	3.75

Water Solubility	
Log *S* (ESOL)	-5.82
Solubility	7.72e-04 mg/ml ; 1.53e-06 mol/l
Class	Moderately soluble
Log *S* (Ali)	-6.20
Solubility	3.17e-04 mg/ml ; 6.28e-07 mol/l
Class	Poorly soluble
Log *S* (SILICOS-IT)	-10.11
Solubility	3.94e-08 mg/ml ; 7.80e-11 mol/l
Class	Insoluble

Pharmacokinetics	
GI absorption	Low
BBB permeant	No
P-gp substrate	Yes
CYP1A2 inhibitor	No
CYP2C19 inhibitor	Yes
CYP2C9 inhibitor	Yes
CYP2D6 inhibitor	Yes
CYP3A4 inhibitor	Yes
Log K_p (skin permeation)	-6.58 cm/s

Druglikeness	
Lipinski	Yes; 1 violation: MW>500
Ghose	No; 2 violations: MW>480, MR>130
Veber	Yes
Egan	Yes
Muegge	Yes
Bioavailability Score	0.55

Medicinal Chemistry	
PAINS	0 alert
Brenk	0 alert
Leadlikeness	No; 2 violations: MW>350, XLOGP3>3.5
Synthetic accessibility	4.00

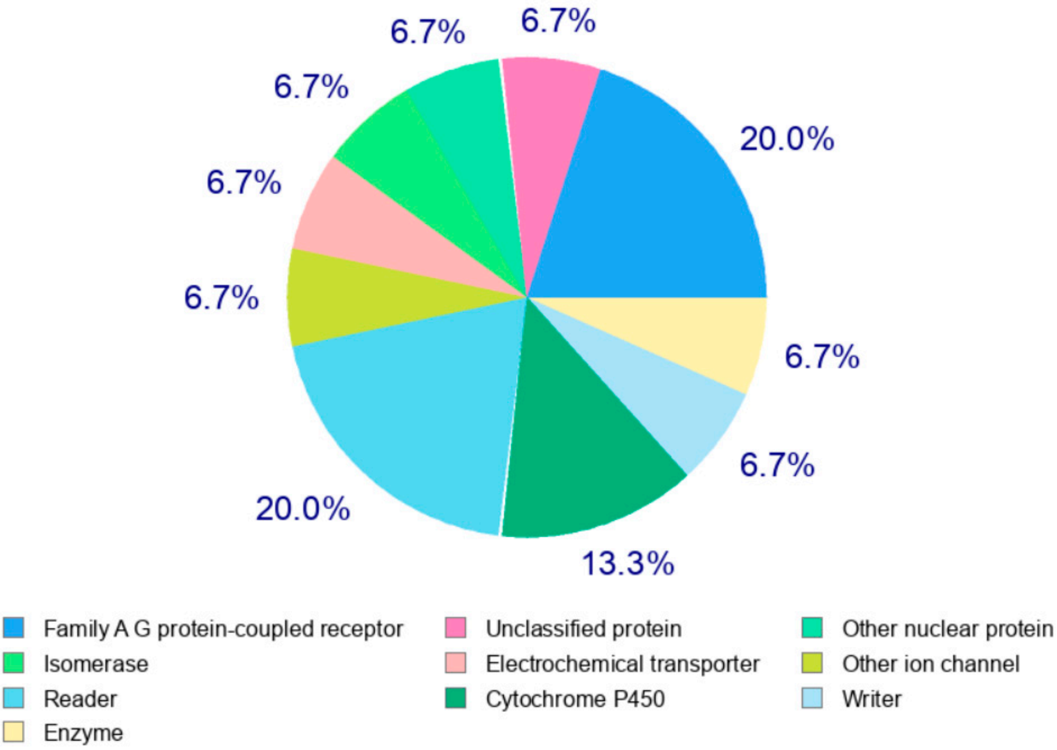

Supplement: Supplementary file 1 [file pharmaceutics-16-01235-s001.zip › pharmaceutics-3189468-supplementary.pdf]
